# Network analysis of canine brain morphometry links tumour risk to oestrogen deficiency and accelerated brain ageing

**DOI:** 10.1038/s41598-019-48446-0

**Published:** 2019-08-29

**Authors:** Nina M. Rzechorzek, Olivia M. Saunders, Lucy V. Hiscox, Tobias Schwarz, Katia Marioni-Henry, David J. Argyle, Jeffrey J. Schoenebeck, Tom C. Freeman

**Affiliations:** 1 Royal (Dick) School of Veterinary Studies and Roslin Institute, University of Edinburgh, Easter Bush Campus, Roslin, Midlothian EH25 9RG UK; 2 Centre for Clinical Brain Sciences, University of Edinburgh, Chancellor’s Building, Edinburgh, Midlothian EH16 4SB UK; 3 Present Address: Medical Research Council Laboratory of Molecular Biology, Cambridge Biomedical Campus, Francis Crick Avenue, Cambridge, CB2 0QH UK; 4 Alzheimer Scotland Dementia Research Centre, University of Edinburgh, 7 George Square, Edinburgh, Scotland EH8 9JZ UK; 5 Present Address: Department of Biomedical Engineering, University of Delaware, Newark, DE 19716 USA

**Keywords:** Cancer epidemiology, Predictive markers, Neural ageing, Brain, CNS cancer

## Abstract

Structural ‘brain age’ is a valuable but complex biomarker for several brain disorders. The dog is an unrivalled comparator for neurological disease modeling, however canine brain morphometric diversity creates computational and statistical challenges. Using a data-driven approach, we explored complex interactions between patient metadata, brain morphometry, and neurological disease. Twenty-four morphometric parameters measured from 286 canine brain magnetic resonance imaging scans were combined with clinical parameters to generate 9,438 data points. Network analysis was used to cluster patients according to their brain morphometry profiles. An ‘aged-brain’ profile, defined by a small brain width and volume combined with ventriculomegaly, was revealed in the Boxer breed. Key features of this profile were paralleled in neutered female dogs which, relative to un-neutered females, had an 11-fold greater risk of developing brain tumours. Boxer dog and geriatric dog groups were both enriched for brain tumour diagnoses, despite a lack of geriatric Boxers within the cohort. Our findings suggest that advanced brain ageing enhances brain tumour risk in dogs and may be influenced by oestrogen deficiency—a risk factor for dementia and brain tumours in humans. Morphometric features of brain ageing in dogs, like humans, might better predict neurological disease risk than patient chronological age.

## Introduction

The global burden of neurological disease has dramatically increased in the last 25 years, largely due to an ageing human population—a trend mirrored in companion animals^1,2^. Much overlap exists between humans and domestic dogs with respect to age-linked vascular, degenerative, and neoplastic brain disorders. Shared environmental influences between these species, as well as the shorter lifespan and refined genetic architecture of pedigree dogs, has driven canines to the leading edge of comparative neurological disease modeling^3–6^.

Brain ageing varies among humans, and biological (physiological) ‘brain age’ better predicts disease risk than chronological age^7–13^. These divergent ageing trajectories might be accentuated in the domestic dog, where selective breeding has produced extreme phenotypic diversity among pedigrees, and where breed plays a role in longevity and the onset of some age-related brain pathologies^14–18^. Emerging evidence points to an increased risk of disease and mortality in humans with structurally ‘older’-appearing brains—dementia, epilepsy, and schizophrenia have all been associated with this enhanced ‘brain age’^8–10,12,13,19–22^. Robust biomarkers of brain ageing are therefore of urgent clinical interest to identify individuals that deviate from a healthy ageing trajectory, enabling targeted early intervention^8–10^.

Certain brain morphometric parameters are predicted to change with neural decline^8,9,16,18,23–41^. However, age-related structural changes are subtle, non-linear, and non-uniform in their distribution^8,10,42,43^. Whilst a single measure is clinically convenient, it is unlikely to capture a phenotype for the complex biological process of ageing^8–10^. Machine learning techniques that estimate ‘brain age’ from human magnetic resonance imaging (MRI) data rely on the fact that morphometric correlates of brain ageing vary little between healthy individuals^8^. This cannot be presumed in the dog, where breed morphometric variations present computational and statistical challenges^5,14,44,45^. Isolating allometric (size-dependent) and non-allometric shape variation is problematic^46,47^, and whilst automated MRI atlas-based protocols have emerged to assess canine brain morphometry^44,45^, their accuracy remains questionable for dogs with structural brain disease and different craniofacial morphologies^5,29,44^. These morphologies—brachycephalic, mesocephalic, and dolichocephalic (‘short-headed, medium-headed, and long-headed’, respectively)—can impact as much on brain shape, as they do on external features of the head^14,44^.

Recent studies have addressed the phenotypic diversity of the domestic dog^15,44,45,48–53^, but the morphometric diversity of the canine brain in a clinical context remains unexplored. Clinical datasets offer several advantages, not least that the natural progression of disease can be observed on a background of both individual- and breed-based heterogeneity. An obvious challenge in exploiting such data is its complexity. To address this issue, we have employed correlation-based network analysis, an unbiased, data-driven method used originally for analysis of transcriptomics data^54–56^, and more recently to explore patient parameters associated with complex syndromes^57^. A key attraction of network analysis is that it incorporates interactions within and between traits—as shown for behavioural phenotypes in dogs^58^. Moreover, network analysis can test previous assumptions made about disease mechanisms and the clinical significance of patient-derived observations^57,59^.

In this study, we have applied network correlation analysis to a complex canine neurological dataset to explore how MRI-based brain morphometry profiles vary according to patient demographics and diagnosis. Our objective was to test statistically for co-enrichment between patient factors, clinical data, and brain morphometric features to extract novel insights into neurological disease risk.

## Methods

### Experimental design

The study plan was to conduct a large-scale, unbiased, hypothesis-generating analysis of complex patient data to identify factors that best predict neurological disease risk in dogs. The Royal (Dick) School of Veterinary Studies Hospital for Small Animals data management system was screened for canine brain MRI scans performed between July 2009 and March 2017. The start date was dictated by MRI availability, and the end point when a minimum of 300 brain scans had been scheduled. Inclusion criteria were MRI of the whole brain, with at least one transverse and one sagittal sequence (T1- or T2-weighted; T1w or T2w), and accessible clinical history. Patients with any trauma or procedure that would alter skull or brain morphometry were excluded. MRI scans were anonymized prior to blinded, quantitative data collection by one of two independent observers (observer A, O.M.S. and observer B, N.M.R.) using the same measurement protocol. Analysis of 47 prospective scans (that met inclusion criteria) were used only to assist with craniofacial category assignment by craniofacial ratio (CFR), assessed by observer B.

### Animals and ethics statement

MRI data were acquired from canine patients as part of routine diagnostic work-up. All patients had been referred to the Hospital and were assessed under the supervision of Board-certified specialists in Small Animal Internal Medicine and/or Neurology. Dogs were anaesthetized and scanned under the supervision of Board-certified specialists in Anaesthesia and Diagnostic Imaging, respectively. Written informed consent of each dog owner was obtained for all diagnostic procedures and for the use of anonymized clinical and imaging data for research purposes.

### Data acquisition

Categories of patient data used for this study are detailed in Supplementary Figs S1–S3. Body weight (kg) was extracted from the anaesthetic record on the day of MRI acquisition. Age was calculated using the date of birth and date of MRI acquisition. Meta-data (sex, breed, category of neurological diagnosis) were extracted using the clinical history, MRI report, clinical pathology reports, final neurologist report, and (where available) histopathology reports. ‘Breed group’ categories were assigned according to the UK Kennel Club registration system (http://www.thekennelclub.org.uk); mixed breed dogs, and those without official breed recognition were either designated a ‘Crossbreed’ grouping or grouped according to the main contributing breed (e.g. Patterdale Terrier = Terrier; Collie X = Pastoral; Beagle X, Whippet X = Hound). Patients were assigned to one of nine diagnostic categories (Supplementary Fig. S1). Anomalous conditions included Chiari-like malformation, syringomyelia, and hydrocephalus; inflammatory conditions were immune-mediated or infectious. The few dogs with degenerative myelopathy and normal brain MRIs were assigned a degenerative diagnosis. A ‘normal’ diagnostic category was assigned only in dogs with structurally normal brains where no neurological diagnosis was made (in these cases, brain MRI was used to rule out a frontal lobe lesion as an explanation for new-onset behavioural changes, where all other clinical tests had failed to reach a diagnosis). Brain morphometric features were measured using OsiriX Medical Imaging Software, and included previously published parameters and recognized normalization factors (Supplementary Fig. S4). CFRs were derived using a modified version of the method described by Packer *et al*.^60^ in which muzzle length (non-linear distance from dorsal tip of nasal planum to the stop in mm) is divided by cranial length (non-linear distance from occipital protuberance to the stop in mm). Measurements and precise locations of the nasal planum, stop, and occipital protuberance were determined on mid-sagittal T2w images using the ‘open polygon’ tool of OsiriX and excluded obvious skin folds. Craniofacial categories were assigned to each patient based on (i) the CFR where available; i.e. where the dorsal tip of the nasal planum was included in the imaging field, (ii) the average CFR recorded for that patient’s breed within the cohort (for purebred dogs, and if there were more than two representatives of the breed with measurable CFRs) and/or (iii) the cut-offs for craniofacial category assignment within our cohort (brachycephaly was defined as a CFR of ≤0.52, mesocephaly as >0.52 to <0.67, and dolichocephaly as ≥0.67). Overall, 139 scans were evaluated by observer A and 172 scans by observer B, with an overlap of 25 scans to evaluate reproducibility of the measurement protocol. Measurements between observers were highly reproducible (variance <10%) for eight parameters; for the remainder, scans measured by observer A were re-measured blind by the more experienced observer B (a board-eligible veterinary neurologist), before processing of the dataset for network analysis (Supplementary Fig. S4e). For scans evaluated by both observers, only data extracted by observer B were used for subsequent analysis.

### Data processing

Raw (measured) data were processed prior to further analysis; brain length, cerebellar volume^61^, cerebellar diameter, interthalamic adhesion height, corpus callosum thickness, and ventricular parameters were normalized to total brain volume (which included ventricular volume)^62^. Cranial length, brain width, total brain volume, and sulcus depth were normalized to body weight to control for allometric scaling^62^. Cerebellar compression length, cerebellar compression index and obex position were normalized to head angle to control for patient positioning. Corpus callosum angle was not normalized. Normalized total brain volumes were retained within the dataset for network analysis but head angle was excluded. Measured ventricle height created a markedly skewed data set due to the recorded ‘zero’ value in most patients. These measurements were therefore categorized to indicate visual integrity of the septum pellucidum: 0 mm = ‘intact’, >0 mm < 3 mm = ‘minor’ loss, >3 mm < 6 mm = ‘moderate’ loss, >6 mm < 10 mm = ‘severe’ loss, >10 mm = ‘absent’. ‘Septal integrity’ thus became an additional meta-data parameter. Age at MRI was categorized as follows: >0 < 2 y = ‘Immature’, ≥2 < 4 y = ‘Young adult’, ≥4 < 8 y = ‘Middle-aged’, ≥8 < 10 y = ‘Mature’, ≥10 y = ‘Geriatric’. Magnitude of variance differed greatly between morphometric measurements, with the potential to disproportionately bias clustering of dogs according to the impact of one or a few parameters. To ensure fair representation of all parameters within the correlation analysis, all numerical data were median-centered for each parameter.

### Network analysis

Normalized, scaled and categorized data were imported into Graphia Professional (Kajeka Ltd., Edinburgh UK), a network analysis software package that calculates data matrices, supports graphical clustering, performs enrichment analyses and identifies patterns in large, complex datasets. The software was originally developed for the analysis of gene expression data, in which the correlation coefficient serves as a measure of co-expression between gene profiles and is used to define edges in a correlation network^54^. For this study, a Pearson correlation was chosen to measure similarity between individual MRI scans based on normalized global brain morphometry measurements. The network graph created from the data was based on a user-defined correlation threshold of *r* = 0.7. This threshold was chosen to incorporate the maximum number of nodes (patient scans) with a minimum number of edges (correlations between patient scans). The measurements of thirteen animals in the cohort shared no correlation with other animals above this threshold and were absent from the graph. Network topology was determined by the number of correlations > *r* = 0.7 between all scans. The Markov clustering (MCL) algorithm^57^ was used to subdivide the graph into discrete clusters of canine MRI scans sharing similar brain morphometric features. Granularity of the clustering (cluster size) is determined by the inflation value (MCLi). For this study, MCLi was set at 2.2 (smallest cluster size of three nodes). A detailed description and validation of the MCL algorithm can be found elsewhere (http://micans.org/mcl)^63^.

### Enrichment and statistical analysis

Graphia Professional’s enrichment analysis uses Fisher’s exact test to determine the probability of a cluster’s composition occurring purely by chance, and offers tools to statistically confirm enrichment of a particular class. Since the canine brain data contained several classes for each MRI scan, Fisher’s exact was used to test each cluster for a disproportionately high representation of each class descriptor. Enrichment outputs include a heatmap and table providing the observed and expected number of members of each class descriptor within each cluster. The corresponding adjusted Fisher’s *P*-value represents how statistically unlikely it is for a class descriptor to occur within a cluster; the lower this value, the more significant the result, and the more brightly it is displayed on the heatmap. All other analyses were conducted in GraphPad Prism 7.0. For comparisons between three or more groups of data, one-way analysis of variance (ANOVA; with Tukey’s multiplicity correction) or Kruskal-Wallis (with Dunn’s multiplicity correction) tests were applied as appropriate, based on data distributions. For comparisons between two data groups, unpaired two-tailed Mann-Whitney, or unpaired two-tailed t-tests (with Welch’s correction if indicated by F-test) were applied. Linear regressions tested for significance between lines of best fit, and Fisher’s exact test was used to assess odds ratios.

## Results

### Complexity within a canine referral cohort

**Figure 1:**
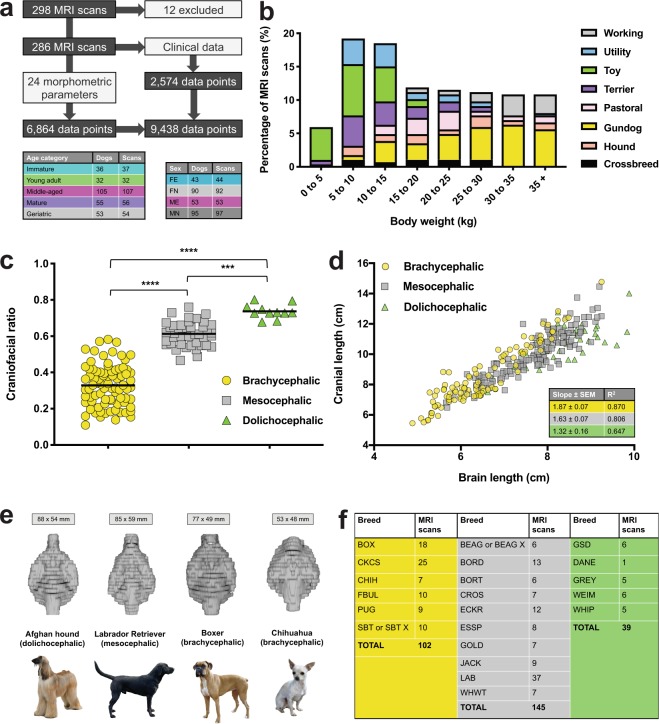
A diverse and complex canine neurological cohort. (**a)** Canine brain MRI scans; 12 were excluded due to lack of required MRI sequences; of the remaining 286 scans, 255 were from pure-bred dogs. Numbers of individual patients and MRI scans included in network analysis are tabulated. Five patients underwent two scans on separate dates. **(b)** Percentage of MRI scans by body weight according to breed group. **(c)** Measurable CFRs (screened from 333 MRI scans). Difference between group means was significant (*N* = 134; *P* < 0.0001; one-way ANOVA; horizontal bars represent mean values and asterisks refer to multiplicity adjusted *P*-values by Tukey’s method ****P < 0.0001 ***P = 0.0007). **(d)** Linear regression of cranial length versus brain length according to craniofacial category. Differences between slopes were significant (*P* = 0.01 brachycephalic versus mesocephalic, *P* = 0.0007 brachycephalic versus dolichocephalic, *P* = 0.04 mesocephalic versus dolichocephalic); SEM = standard error of the mean. **(e)** Computed brain volumes for representative dolichocephalic, mesocephalic, and both large and small brachycephalic breeds; mean brain lengths and widths are shown for each breed. **(f)** Numbers of MRI scans used in network analysis for breeds with ≥ five representatives, by craniofacial category (brachycephalic, yellow; mesocephalic, grey; dolichocephalic, green). BOX (Boxer), CHIH (Chihuahua), FBUL (French Bulldog), PUG (PugDog), SBT (Staffordshire Bull Terrier), BEAG (Beagle), BORD (Border Collie), BORT (Border Terrier), CROS (Crossbreed), ECKR (English Cocker Spaniel), ESSP (English Springer Spaniel), GOLD (Golden Retriever), JACK (Jack Russell Terrier), LAB (Labrador Retriever), WHWT (West Highland White Terrier), GSD (German Shepherd Dog), DANE (Great Dane), GREY (Greyhound), WEIM (Weimeraner), WHIP (Whippet). Canine breed image attributions: AFGH (By SheltieBoy (Flickr: AKC Helena Fall Dog Show 2011) [CC BY 2.0 (https://creativecommons.org/licenses/by/2.0)]); BOX (By Flickr user boxercab (Flickr here) [CC BY 2.0 (https://creativecommons.org/licenses/by/2.0)]); CHIH (Photo taken by en:User:Exdumpling in 2004 and uploaded to English Wikipedia as WhiteTanChihuahua.jpg claiming own work with PD-self license); LAB (By Desaix83, d’après le travail de Chrizwheatley [Public domain]).

A total of 9,438 morphometric and clinical data points were extracted from 286 MRI scans conducted on 281 individual dogs (Figs 1a, S1). These included 61 UK Kennel Club breeds and all seven recognized Kennel Club breed groups (Supplementary Fig. S2, Supplementary Data File S1). The most common breeds in the cohort were Labrador Retriever (12.9%), Cavalier King Charles Spaniel (CKCS; 8.7%), and Boxer (6.3%); 52.7% of scans derived from male dogs and 65.8% of patients were neutered. Median age at MRI was 6.8 y (range 0.2–17.4 y) and median body weight was 18.1 kg (range 1.2–97.0 kg). The distribution of body weights and ages according to breed grouping highlighted the diversity within our cohort (Figs 1b, S3). Measurements to determine CFRs^60^ were possible in 117 retrospective scans, and in 17 of 47 prospective scans used to support craniofacial category assignment (Figs 1c, S2b, S4, Supplementary Data File S2). Based on defined cut-offs, 35.7% of MRI scans used for network analysis derived from brachycephalic, 50.7% from mesocephalic, and 13.6% from dolichocephalic dogs. Brachycephalic dogs had shorter brains relative to their cranial length and were predominantly found within Toy, Utility and Working groups (Figs 1d–f, S3d). Mesocephalic and dolichocephalic dogs were mainly found within Gundog and Hound groups, respectively. The distribution of MRI scans across craniofacial categories according to genetic clade^64^ (Supplementary Fig. S5) identified a large contribution of European Mastiff, Retriever, and UK Rural clades to brachycephalic, mesocephalic, and dolichocephalic MRI scans, respectively. Overall, our cohort reflected the complex demographic of canines referred to neurology, on a background of current breed preferences among UK dog owners.

### Network analysis reveals clustering of canine brains

**Figure 2:**
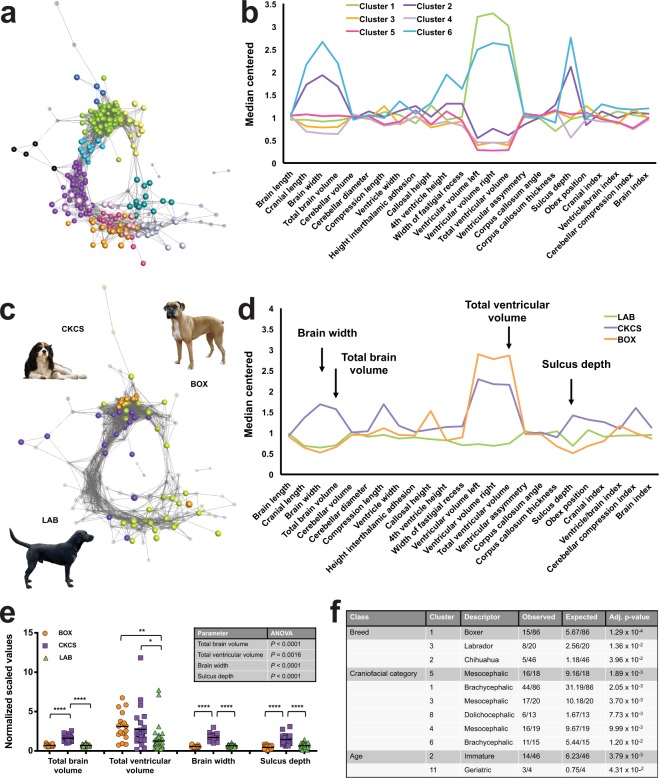
Network analysis reveals clustering of canine brains based on morphometry. In the network, nodes represent individual MRI scans; edges represent Pearson correlation coefficients (*r* > 0.7) between their brain morphometry profiles. Non-clustered and unselected nodes are displayed as smaller transparent spheres. Some nodes are hidden within clusters or on other aspects of the graph; iterations of the network can be explored by inputting Supplementary Data File S3 into Graphia Professional. **(a)** Network with nodes coloured by cluster; median lines for the six largest clusters are shown in associated chart **(b)**. Note that sulcus depth, ventricular volume, and whole brain parameters (length, width, volume) drive divergence of canine brain morphometry profiles. **(c**–**e)** Brain morphometry comparison for three most common breeds in the cohort. Arrows in **(d)** indicate key morphometric parameters tested in **(e)** (*N* = 79). Differences between group means were significant as shown in the inset table (one-way ANOVA); in the dot plots, horizontal bars represent the mean value and asterisks refer to multiplicity adjusted *P*-values by Tukey’s method (****P < 0.0001, ***P < 0.001). **(f)** Enrichment analysis of breeds, craniofacial categories and age categories within clusters. Enrichments are listed only where observed node numbers were ≥ three (minimum cluster size). Note strong enrichment of brachycephalic Boxer dogs in cluster one. Canine breed image attributions: BOX (By Flickr user boxercab (Flickr here) [CC BY 2.0 (https://creativecommons.org/licenses/by/2.0)]); CKCS (By Mário Simoes (Flickr: Cavalier King Charles Spaniel) [CC BY 2.0 (https://creativecommons.org/licenses/by/2.0)]); LAB (By Desaix83, d’après le travail de Chrizwheatley [Public domain]).

**Figure 3:**
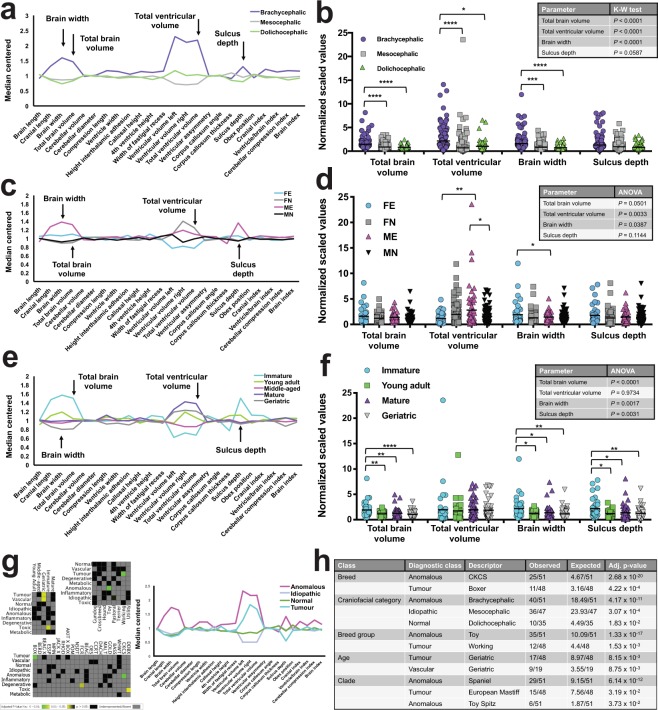
Signalment and diagnosis impact on canine brain morphometry. Brain morphometry comparisons by (**a**,**b)** craniofacial category, **(c**,**d)** sex, and **(e**,**f)** age category. Arrows in **(a**,**c**,**e)** indicate key morphometric parameters tested in **(b)** (*N* = 286), **(d)** (*N* = 286), and **(f)** (*N* = 179) by one-way ANOVA or Kruskal-Wallis (K-W) test as required. Differences between group means or medians (horizontal bars) are shown in each inset table, with significance depicted in shaded boxes; asterisks refer to multiplicity adjusted *P*-values by Tukey’s or Dunn’s method (****P < 0.0001, ***P < 0.001, **P < 0.01, *P < 0.05). In **(c)** and **(d)** FE = un-neutered females, FN = neutered females, ME = un-neutered males, and MN = neutered males. **(g)** Heat maps and chart coloured by final neurological diagnosis. Note enrichment of tumour diagnoses with both the geriatric group and Boxer breed. Network graphs for each diagnostic class are visualized separately in Supplementary Figure S10. **(h)** Enrichment analysis results for diagnostic class sets. Table lists significant enrichments together with expected and observed numbers for each descriptor that occurred in a given class, with adjusted *P*-values. Enrichments were excluded where observed number of nodes was < three (minimum cluster size). For each class, descriptors are listed in order of statistical significance. No enrichments were found within diagnostic class sets for septal integrity or sex.

At a correlation threshold of *r* = 0.7, a graph was generated incorporating 273 MRI scans (nodes) and 3,911 correlations (edges) (Figs 2a, S6, Supplementary Data File S3). The graph’s topology exhibited distinct cliques (areas of high connectivity) and with MCL produced 12 clusters, incorporating 250 scans (Supplementary Fig. S7, Supplementary Table S1). Patients within each cluster shared similar brain morphometric features, with 71.3% of scans residing in one of six large clusters (Fig. 2b). Figure 2c–e compare the brain morphometry profiles of the three most common breeds within the network; CKCS dogs were distinguished by their ventricular parameters and cerebellar compression, and Boxer and Labrador brains diverged mainly on the basis of ventricular size. To evaluate the statistical significance of cluster composition, an enrichment analysis was performed for each cluster of data (Fig. 2f). Cluster one was enriched for brachycephalic Working dogs (including 14 Boxers). Immature and Chihuahua dogs were over-represented in cluster two, whilst cluster three was enriched for mesocephalic Gundogs including Labradors. Cluster four featured mesocephalic Crossbreed dogs, and mesocephalic dogs were also over-represented in cluster five. Dolichocephalic and geriatric dogs were enriched in clusters eight and eleven, respectively. Overall, relative to other craniofacial categories, brachycephalic dogs had larger brain widths, enlarged ventricular parameters, and greater cerebellar compression (Figs 3a,b, S8). Conversely, dolichocephalic dogs had narrower brains with intermediate ventricular volumes, and mesocephalic dogs had small ventricular volumes. Variation was observed in brain morphometry profiles according to sex; un-neutered animals had larger brains relative to their body weight, although this was offset by an increased ventricular size and sulcus depth in males (Fig. 3c,d). Neutered and un-neutered females had the largest and smallest ventricular volumes, respectively. Whole brain parameters (length, width and volume), ventricular size, sulcus depth, and corpus callosum thickness separated the youngest and oldest dogs (Fig. 3e–f). In summary, signalment (breed, craniofacial category, sex, and age) appeared to drive the clustering of canine brains, with ventricular size and brain width being most impacted by these factors.

### Clinical-morphometric interactions identify the Boxer as an outlier

**Figure 4:**
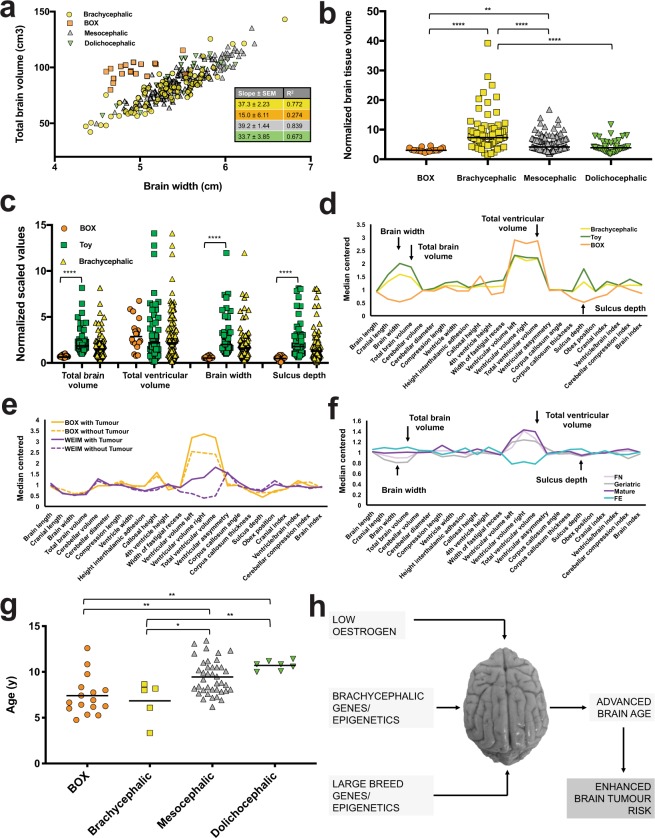
Advanced ‘brain age’ linked to tumour risk and oestrogen loss. (**a)** Linear regression of total brain volume versus brain width; differences between Boxer and other slopes were significant (*P* = 0.004 other brachycephalic, *P* = 0.0001 mesocephalic, *P* = 0.02 dolichocephalic); SEM = standard error of the mean. **(b)** Residual brain tissue volume (ventricular volume subtracted from total brain volume) normalized to body weight. Difference between group medians (horizontal bars) was significant (*N* = 286, *P* < 0.0001; Kruskal-Wallis); asterisks refer to multiplicity adjusted *P*-values by Dunn’s method (****P < 0.0001). **(c**,**d)** Total brain volume, brain width, and sulcus depth (arrowed) appear small in the Boxer relative to other brachycephalic breeds; horizontal bars in **(c)** represent median values and asterisks refer to statistical significance Mann-Whitney test (*N* = 72, ****P < 0.0001). Note that statistical comparisons with the brachycephalic group are not made since, in this instance, Boxers (and also some Toy breeds) are included within the brachycephalic group. **(e)** The Boxer brain morphometry profile is not explained by tumour growth, which in another large breed (Weimeraner) has a marked impact on ventricular size and network position (Supplementary Fig. S13g). **(f)** Brain morphometry of neutered female dogs mimics that of mature and geriatric dogs, with a significantly larger ventricular volume than in un-neutered females (*N* = 136, *P* = 0.004; unpaired two-tailed t-test with Welch’s correction). **(g)** Age distribution of patients diagnosed with brain tumours since the start of the study period by craniofacial category. Difference between group means (horizontal bars) was significant (*N* = 67, *P* < 0.0001; one-way ANOVA); asterisks refer to multiplicity adjusted *P*-values by Tukey’s method (**P < 0.01, *P < 0.05). **(h)** Proposed model for factors contributing to advanced brain age and brain tumour risk in dogs. Photograph of canine brain contributed by lead author.

Observing that some diagnostic classes were prominent among certain demographic categories and clusters (Fig. 3g, Supplementary Figs S9–10), we next explored correlations between signalment, brain morphometry, and neurological disease. Interestingly, cluster one contained 26 dogs with tumour diagnoses (ten of which were Boxers) and there were patients in all breed groups with ‘idiopathic’ diagnoses based on clinical signs and a normal MRI—many of these had epilepsy. The Fisher’s exact test was used to detect enrichment of signalment descriptors within each diagnostic class (Fig. 3h). Significant enrichments included brachycephalic dogs within the anomalous class, whilst geriatric dogs were enriched within tumour and vascular classes. Four out of ten Pointer Setter dogs had brain tumours, and three of these were neutered female geriatric Weimeraners (age and breed co-enriched with adjusted *P*-value of 2.38 × 10^−2^). Boxer dogs were greatly enriched within the tumour class, and mesocephalic dogs were over-represented within the idiopathic class. With respect to breed group, the anomalous class was significantly enriched for Toy dogs (mainly CKCS reflecting the high prevalence of Chiari-like malformation in this breed)^65^, whilst the tumour class was enriched for Working group dogs (mainly Boxers). Again, ventricular size strongly dictated clustering and group dynamics; four out of seven Labrador Retrievers positioned in cluster one had tumours and large ventricular parameters. Working and Toy breeds had the largest ventricular volumes, but these breed groups dramatically diverged with respect to whole brain parameters and sulcus depth. Strikingly, Boxers had remarkably narrow brains (Fig. 4a), accentuating a feature more consistent with a dolichocephalic phenotype (Fig. 3a). Moderate to severe loss of the septum pellucidum (membrane that separates the lateral ventricles of the brain) was prominent in the European Mastiff clade, which was also enriched for entire male dogs (adjusted *P*-value 9.55 × 10^−3^). Septal integrity was most compromised in the Boxer; only five out of 18 dogs had a visually intact septum (Supplementary Fig. S11). Combined with ventriculomegaly, the reduced whole brain dimensions in the Boxer resulted in a small residual brain tissue volume relative to body size, a feature which clearly separated the Boxer from other brachycephalic breeds (Fig. 4b). Together, our results defined the Boxer as an outlier, displaying both brachycephalic and dolichocephalic morphometric features, alongside an increased tumour risk.

### Advanced ‘brain age’ in the Boxer and neutered female dogs

Having confirmed enrichment of the Boxer breed with tumours, but not with the geriatric class (despite geriatric scans being enriched for tumours), we considered that Boxer brains may be subject to accelerated ageing. Indeed, Boxer brain morphometry profiles exaggerated those of mature and geriatric dogs (Fig. 3e). Apart from ventriculomegaly, the ‘aged’ Boxer profile did not broadly represent the brachycephalic phenotype (Fig. 4c,d). Boxer brain morphometric features were shared with some other members of the Working group (Rottweiler and Dogue de Bordeaux), and European Mastiff clade (Boston Terrier and Rhodesian Ridgeback; Supplementary Fig. S12), but not all representatives (French Bulldog and Staffordshire Bull Terrier). To confirm that Boxer brain morphometry did not simply reflect tumour growth, the profiles of breeds with a high risk of tumours in our cohort (Boxer and Weimeraner) were compared, in the presence and absence of tumour diagnoses (Fig. 4e). In the Boxer, tumour diagnosis was associated with a marginal increase in ventricular size, whereas in the Weimeraner, it converted a small ventricular profile to one consistent with ventriculomegaly. Follow-up scans in five dogs exposed the dynamism of cerebrospinal fluid (CSF)-filled spaces in response to partial or complete resolution of brain lesions (Supplementary Fig. S13). However, the ‘aged’ morphometry profile appeared unique to the Boxer and was retained both before and after treatment. Intriguingly, the aged Boxer profile mimicked that of neutered females (Fig. 4f), which had a high proportion of tumour diagnoses (21.1%) relative to un-neutered females (4.6%; the lowest percentage of the four sex categories within the network). By contrast, un-neutered females had large whole brain parameters and small ventricles (*P* = 0.004, unpaired two-tailed t-test with Welch’s correction, relative to neutered females), and enriched with immature brain profiles (*P* = 8.97 × 10^−4^; Supplementary Fig. S14). Critically, although neutered females were on average older than un-neutered females in our cohort, the relative increase in the size of their ventricles was significant in the geriatric group (Supplementary Fig. S15). Within the network, seven of ten Boxer dogs with tumours were middle-aged. Prospective analysis of 148 dogs presenting for brain MRI at our institution identified an additional 23 dogs with brain tumours, all of which were entire males or neutered animals, including four Boxers with a mean age of 7.5 y. Boxers and other brachycephalic dogs were thus diagnosed earlier with brain tumours than other breed types (Fig. 4g). Finally, considering all brain scans performed to date (441 in 429 dogs), and excluding tumours that had metastasized from other parts of the body to the brain (Supplementary Data File S4), neutering increased the relative risk of brain tumours 11-fold in females (odds ratio 13.5, *P* = 0.0006, 95% confidence interval 2.4-141.4), and un-neutered females were seven times less likely to suffer brain tumours than un-neutered males (odds ratio 7.5, *P* = 0.03, 95% confidence interval 1.3-82.4). In conclusion, our findings suggest that oestrogen may be protective against brain ageing and brain tumour growth in dogs, whereas the Boxer is at high risk for both (Fig. 4h).

## Discussion

Applying a data-driven approach, we have identified an aged-brain morphometric phenotype in Boxers and neutered female dogs that enriches with brain tumour risk. To our knowledge, this is the first network analysis of global brain morphometry, and the first study to link brain tumour risk to structural brain ageing. Our results are consistent with the hypothesis that structural ‘brain age’ influences disease risk, and that oestrogen plays a role in brain ageing and tumour growth.

All canine patients underwent MRI because of brain-localising signs, therefore our cohort does not represent healthy brain ageing. Combining idiopathic and normal (undiagnosed) patients, our network included 31.3% structurally ‘normal’ brains. Subtle morphometric changes (Supplementary Fig. S16) caution against describing idiopathic epileptic brains as ‘normal’^66^, although others have used epileptic patients to establish reference values in dogs^34^. Some tumour diagnoses were not confirmed because a necropsy was not performed (Supplementary Data File S4), although other major differentials were ruled out with CSF analysis. Manual planimetry techniques are arguably more precise than semi-automated approaches^42^, and the measurement protocol used for this study had a similar time commitment to that reported with atlas-based segmentation (around 30 min per brain)^44^. However, given the time and effort required by trained clinical staff to extract the data, we do not advocate the use of this protocol for routine clinical application; rather we highlight the value of applying network analysis to complex clinical datasets. Whilst templates derived from a small number of breeds without structural brain pathology enable reproducible morphometric analysis^29,44,45,67^, these templates rapidly become inaccurate in the face of structural brain disease^44^. Milne *et al*., attempted to account for craniofacial diversity in their development of brain atlas templates, however the patients used to generate these templates included neurologically abnormal dogs (with ataxia, vestibular disease, and idiopathic cerebellitis) and the assignment of craniofacial category was subjective^44^. The authors argue that *‘subjective evaluation allows for the formation of a global opinion by taking into account a complex array of volumetric factors and spatial relationships’*; by contrast, we have taken objective measurements and then used network analysis to extract an unbiased view of the complex relationships between them. Importantly, this approach is readily applicable to datasets extracted by manual or automated methods. Referral bias will have magnified enrichments for breeds that most frequently present to our institution; network analysis partly controls for this issue, but it cannot eliminate the need for larger datasets to model disease risk at the population level.

A 40-fold difference in skeletal size exists between the largest and smallest dog breeds, and there is a strong correlation between body weight and the volume of several brain compartments^34,50,62,68,69^. Most parameters were normalized to total brain volume to help control for individual and breed variations in brain morphometry^25,30,70^. Some have argued against using ventricular-to-cerebrum ratio to assess brain ageing since such measures would be breed-specific^62^, yet this presumes that ventriculomegaly reflects normal breed variation. Boxers without cerebral disease have large lateral ventricles relative to other breeds^71^, however the definition, development, and clinical significance of ventriculomegaly in dogs remains controversial^72^. Ageing has been associated with changes in brain and ventricular volume in dogs, but most data comes from laboratory Beagles^16,18,23,24,26,27,29,30,35,73^. Given the extensive breed variation in canine ventricular morphology, age- and breed-specific reference ranges (obtained from neurologically normal dogs using a standard set of MRI sequences) are needed to determine the relevance of our findings to the broader canine population. Training healthy dogs to participate in advanced neuroimaging studies without anaesthesia may address some ethical concerns and deliver the statistical power required for complex morphometric research questions^5,42^. Current limitations including the potential impact of brain pathology on specific morphometric parameters preclude us from defining a statistical threshold for ventriculomegaly. Likewise, we consider it inappropriate to quantitatively define ‘brain age’ from our retrospective dataset; rather we refer to an ‘aged-brain profile’, reflecting the specific combination of morphometric parameters that characterize this structural brain phenotype in our cohort.

Certain observations built confidence in our analysis, not least the relative ventriculomegaly in brachycephalic breeds, and cerebellar compression in CKCS dogs^65,72,74^. Enrichment of Boxers with tumours was anticipated^74^ and the compromised septal integrity in this breed is more common in brachycephalics generally (29% versus 9% and 13% in mesocephalic and dolichocephalic breeds in our network, respectively). Non-detection of the canine septum pellucidum on MRI is largely considered incidental^70,75,76^, and it remains possible that the septum is intact, but too thin to be observed in some dogs^70^. Apparent absence of the septum has been observed in neurologically normal humans but is often associated with other structural anomalies^70,77^. Interestingly, 21 of 25 CKCS dogs in our cohort had intact septa, despite their high prevalence of Chiari-like malformation^78^. Conceptually, a compromised septum might increase ventricular compliance and thus explain why Boxers are at low risk of Chiari-like malformation and syringomyelia, despite shared ventricular morphology with CKCSs.

The need to explore sexual dimorphism in brain ageing is underpinned by the fact that dementia disproportionately affects women^79^. The largest ever single-sample neuroanatomical study of sex differences using UK Biobank data found several sexually dimorphic differences in human brain structure^42^. Importantly these changes operated in a global manner, supporting our approach to consider multiple morphometric features in concert, and to correct for total brain volume. Age-related structural brain changes differ between men and women^36^, and also between male and female dogs^16,29^. Men exhibit greater increases in sulcal and ventricular CSF volume^36,37^, whilst women demonstrate greater rates of hippocampal atrophy^38–40^. A semi-quantitative visual rating scale was used to chart cerebral involutional changes in dogs, however neither sex nor neutering status were considered as co-variates^23^. One canine study reported that different brain regions appeared more vulnerable to atrophy in males—although these animals were all sexually intact^29^. Post-mortem studies in German Shepherd Dogs found ventricular enlargement with ageing and no apparent relationship to sex, although again the effect of neutering was not explored^27^. By contrast, our results indicate an accelerated ventriculomegaly and total brain loss in neutered female dogs. Importantly, whilst there was a difference in age distribution between sex categories in our network, there was a trend for enhanced ventriculomegaly in neutered females across all age categories in adulthood, reaching significance in the geriatric group. To the authors’ knowledge, this is the first study to demonstrate an effect of neutering status on brain morphometry in dogs.

Oestrogen deficiency is proposed to explain accelerated brain ageing in post-menopausal women as well as an accelerated epigenetic clock in ovariectomized mice^80–82^. Several meta-analyses have shown hormone-replacement therapy (HRT) to be neuroprotective, and although recent publications have raised doubt over the ‘oestrogen deficiency’ theory of dementia^83–85^, HRT may still defend cognition in a subgroup of women in the perimenopausal period^85,86^. Our work supports the concept that oestrogen loss may accelerate neural decline, however causal mechanistic insight is lacking. Development of multicentre canine biobanks will facilitate investigations of oestrogen status as a function of brain ageing in dogs, and whether this relates to cognitive dysfunction. In human patients, advanced structural ‘brain-age’ is often paralleled by epigenetic markers of ageing^9,11,87,88^. The premise that oestrogen may have neuroprotective benefits across the lifespan—and that its effects may be epigenetically regulated^89^—emphasises the need to integrate structural, functional, and molecular approaches in the study of brain ageing and brain disease.

Canine gliomas occur most commonly in brachycephalic breeds, with the Boxer at highest risk^74^. We noted a significant enrichment of brain tumours with Boxers, however the absence of tumour diagnoses in CKCS dogs resulted in non-enrichment of tumours within our brachycephalic category. The majority of Boxers in our cohort were middle-aged, consistent with the findings of Song *et al*. where gliomas most frequently occurred in dogs aged seven-to-eight years^74^. This is despite the fact that increasing age remained a risk factor for all intracranial neoplasias (as seen here)^74^. An increased risk of primary intracranial neoplasms has also been found in large breed dogs^74^, and indeed, only 11 of 52 dogs with tumour diagnoses in our network were small breeds. Most size variation between purebred dogs is controlled by a few genes of major effect, including several members of the insulin-like growth factor-1 (*Igf-1*) pathway^4,46,50^. *Igf-1* is a major determinant of dog size; its variable expression is proposed to underlie the increased longevity of smaller breeds and the higher frequency of neoplasia-associated deaths in large breeds^62^. Coincidentally, the rapid growth of large breeds may initiate premature ageing due to increased free radical release during development^90^. Roughly half of small or medium breed dogs also have ‘large alleles’, mainly found in muscled breeds such as the French Bulldog and Boxer^46,48^. Our analysis reveals that Boxers have ventricular parameters of the small brachycephalic phenotype, but whole brain parameters of large breed mesocephalic or dolichocephalic phenotypes. Conceivably, a combination of variants promoting brachycephaly (e.g. *Smoc2*), on a background of those promoting growth (*Igf-1*) may place the Boxer at extreme risk of premature ageing and brain tumours^48^.

Although primary brain tumours can affect men or women at any age, emerging evidence supports a role for both chromosomal and gonadal sex in neuro-oncogenesis and brain ageing^91^. Malignant gliomas are more common in men globally, and the risk of intracranial tumours is increased in women with complete or partial X-chromosome monosomy and low oestrogen levels^92–94^. The human male predominance for brain tumours appears to persist in all age groups, indicating that acute effects of circulating sex steroids cannot simply explain the sexual disparity in tumour risk^92^. Mosaic loss of chromosome Y, the most common acquired human mutation and another putative biomarker of ageing, has been associated with an increased risk both of Alzheimer’s disease and various cancers^95–97^. To our knowledge, this is the first study to report a reduced risk of brain tumours in un-neutered female dogs relative to neutered animals. Given the routine (but not mandatory) practice of neutering, an unrivalled opportunity exists to explore the influence of chromosomal and gonadal sex on neuropathology in canines of varied neutering status.

Extreme breed characteristics impact on health and welfare, with widespread concerns surrounding brachycephaly^61,90^. Our work extends this to the brain, highlighting an urgency to better understand the factors that influence brain ageing in dogs. Simultaneously, comparative studies will accelerate our knowledge of how chromosomal and hormonal sex affect brain structure, brain ageing, and brain tumour development in humans. The Boxer breed in particular could represent a valuable model of naturally-enhanced brain ageing. Larger, longitudinal imaging studies are required to confirm how patient demographics influence brain age—network analysis can facilitate discovery of subtle yet important phenotypic shifts within these complex clinical datasets. Importantly, our unique application of network analysis can be immediately translated to pre-existing and emerging human patient data. A key question is whether canine brain morphometry and associated morbidity can be explained by selectively-driven changes in skull shape, or whether independent genetic, epigenetic, or epidemiological factors contribute to neurological disease. Isolating these factors will advance our understanding of disease pathogenesis, with important implications for canine and human brain health^3,6^.

## Supplementary information

Supplementary Information

Date File S1

Data File S2

Date File S3

Date File S4

## Data Availability

Anonymised DICOM files are available on request. Supplementary Data Files are deposited at Mendeley Data (10.17632/y2f9272bbd.1). Graphia Professional is free to download at https://kajeka.com/graphia-professional/.
