# Supplementary material for: Network analysis of canine brain morphometry links tumour risk to oestrogen deficiency and accelerated brain ageing
Source: Sci Rep. 2019 Aug 29;9:12506. doi: 10.1038/s41598-019-48446-0 (PMC6715702; doi:10.1038/s41598-019-48446-0)
Supplement: Supplementary file 1 — Supplementary Information [file 41598_2019_48446_MOESM1_ESM.pdf]

Supplementary Information

Network analysis of canine brain morphometry links tumour risk to oestrogen deficiency and accelerated brain ageing

Authors

Nina M. Rzechorzek PhD[1,2,3]*, Olivia M. Saunders BVM&S[1], Lucy V. Hiscox PhD[4,5], Tobias Schwarz DipECVDI[1], Katia Marioni-Henry PhD[1], David J. Argyle PhD[1], Jeffrey J. Schoenebeck PhD[1], Tom C. Freeman PhD[1]

Supplementary Figures

Fig.S1. Patient parameters recorded for each MRI scan

Fig.S2. Breed characteristics for MRI scans

Fig.S3. Age and body weight distribution of dogs in network

Fig.S4. Canine brain morphometric measurement protocol and reproducibility

Fig.S2. Percentage of MRI scans by genetic clade

Fig.S6. Correlation graph settings for Graphia Professional dataset

Fig.S7. Cluster characteristics

Fig.S8. Networks by signalment

Fig.S9. Demographics and enrichment of diagnoses

Fig.S10. Diagnostic class morphometrics

Fig.S11. Septal integrity impacts on brain morphometry

Fig.S12. Shared morphometric features of the Boxer

Fig.S13. Patients scanned twice during the study period

Fig.S14. Enrichments by sex

Fig.S15. Ventricular enlargement in neutered female dogs

Fig.S16. Dichotomous brain morphometry in dogs with and without paroxysmal events

Supplementary Tables

Table S1. Clusters generated for network

Supplementary References

Supplementary Figures

Clinical history data	MRI measurements	Derived from MRI measurements
Unique identifier<br>Date of MRI scan<br>Date of data extraction<br>Clade[1]<br>Breed group§<br>Breed<br>Craniofacial category*<br>Sex<br>Age at MRI (y)<br>Body weight at MRI (kg)[2]<br>Final neurological diagnosis category†	***General skull/brain morphology***<br>Brain (cerebral) length (cm)<br>Cranial length (cm)<br>Brain width (cm)[3]<br>Total brain volume (cm^3)[4]<br>Cerebellar volume (cm^3)[4]<br>Combined sulcus depth (mm)<br>Cranial length nonlinear (mm)<br>Muzzle length nonlinear (mm)<br>***Ventricular morphology and hydrocephalus***<br>Interthalamic adhesion height (mm)[5]<br>Corpus callosum thickness (mm)[6-7]<br>Ventricle width (cm)<br>Callosal height (mm)[6]<br>4th ventricle height (mm)[6]<br>Width of fastigial recess (mm)[6]<br>Volume left lateral ventricle (cm^3)<br>Volume right lateral ventricle (cm^3)<br>Corpus callosum angle (degrees)[3]<br>Ventricle height (mm)[8]<br>***CLM measures***<br>Obex position (mm)[9]<br>Compression length (mm)[10]<br>Cerebellar diameter (cm)[10]<br>***Normalization factors***<br>Head angle (degrees)[3]	Total ventricular volume (cm^3)<br>Integrity of septum pellucidum[5]<br>Cranial index (cranial breadth/cranial length x 100)[3]<br>Cerebellar to total brain volume ratio (x 100)[4]<br>Ventricle/brain index[6]<br>Cerebellar compression index[9]<br>Craniofacial ratio<br>Ventricular asymmetry

Fig.S1. Patient parameters recorded for each MRI scan. §Working, Gundog, Utility, Toy, Terrier, Hound, Pastoral, Crossbreed. *Brachycephalic, mesocephalic or dolichocephalic. †Normal, Tumour, Degenerative, Vascular, Anomalous, Inflammatory (infectious/immune-mediated), Metabolic, Toxic, Idiopathic. Metadata columns (left) and numerical values (middle and right) that were later incorporated into the network analysis are highlighted in red.

a

Breed (Group/clade)	Abbreviation	MRI scans	Breed (Group/clade)	Abbreviation	MRI scans	Breed (Group/clade)	Abbreviation	MRI scans
Boston Terrier (U/EM)	BOST	2	Akita X Boxer* (C/C)	AKIT X BOX	2	Afghan Hound (H/M)	AFGH	2
Boxer* (W/EM)	BOX	18	American Cocker Spaniel (G/Sp)	ACKR	2	Bassett Hound (H/SH)	BASS	2
Brussels Griffon (To/TS)	BRUS	1	Beagle or Beagle X (H/SH)	BEAG or BEAG X	6	German Shepherd Dog (P/NW)	GSD	6
Bulldog (U/EM)	BULD	2	Bearded Collie (P/UR)	BERD	1	Great Dane (W/EM)	DANE	1
Cairn Terrier (Te/T)	CAIR	1	Bichon Frise (To/Po)	BICH	2	Greyhound (H/UR)	GREY	5
Cavalier King Charles Spaniel (To/Sp)	CKCS	25	Border Collie (P/UR)	BORD	13	Italian Greyhound (To/UR)	ITGY	1
Chihuahua (To/AT)	CHIH	7	Border Terrier (Te/T)	BORT	6	Lurcher or Lurcher X (C/C)	CROS	3
Chinese Shar-Pei (U/AS)	SHAR	1	Cockerpoo (C/C)	CROS	1	Miniature Dachshund (H/SH)	DACH	1
Crossbreed (C/C)	CROS	1	Collie or Collie X (P/UR)	COLL or COLL X	4	Miniature Poodle (U/Po)	MPOO	1
Dogue de Bordeaux (W/EM)	DDBX	1	Crossbreed (C/C)	CROS	7	Scottish Deerhound (H/UR)	DEER	1
English Mastiff (W/EM)	MAST	1	Curly Coated Retriever (G/R)	CCRT	1	Shetland Sheepdog (P/UR)	SSHP	1
French Bulldog (U/EM)	FBUL	10	Dalmatian (U/PS)	DALM	2	Siberian Husky (W/AS)	HUSK	1
King Charles Spaniel (To/Sp)	KCS	2	English Cocker Spaniel (G/Sp)	ECKR	12	Standard Poodle* (U/Po)	SPOO	3
Lhasa Apso (U/AsT)	LHSA	1	English Springer Spaniel (G/Sp)	ESSP	8	Weimeraner (G/PS)	WEIM	6
Maltese Terrier (To/Po)	MALT	3	Flat Coated Retriever (G/R)	FCR	2	Whippet* (H/UR)	WHIP or WHIP X	5
Newfoundland (W/R)	NEWF	1	German Shorthaired Pointer (G/PS)	GSHP	1			
Pomeranian (To/SS)	POM	1	Giant Schnauzer (W/D)	GSNZ	1			
PugDog (To/TS)	PUG	9	Golden Retriever (G/R)	GOLD	7			
Shih Tzu (U/AsT)	SHIH	4	Irish Setter (G/PS)	ISET	1			
Staffordshire Bull Terrier or X (Te/EM)	STAF or STAF X	10	Jack Russell Terrier (Te/T)	JACK or JACK X	9			
Yorkshire Terrier (To/T)	YORK	2	Labrador Retriever* (G/R)	LAB	37			
			Miniature Pinscher (To/P)	MPIN	1			
			Miniature Schnauzer (U/S)	MSNZ	3			
			Novia Scotia DTR (G/R)	NSDT	1			
			Old English Sheepdog (P/UR)	OES	1			
			PatterdaleTerrier (Te/T)	CROS	1			
			Rhodesian Ridgeback (H/EM)	RHOD	1			
			Rottweiler (W/D)	ROTT	1			
			Soft Coated Wheaten Terrier (Te/T)	SCWT	1			
			Standard Schnauzer (U/S)	SSNZ	1			
			Terrier X (Te/T)	CROS	1			
			West Highland White Terrier (Te/T)	WHWT	7			

b

Breed (Group/clade)	Abbreviation	MRI scans	Breed (Group/clade)	Abbreviation	MRI scans	Breed (Group/clade)	Abbreviation	MRI scans
Boston Terrier (U/EM)	BOST	3	American Cocker Spaniel (G/Sp)	ACKR	2	Afghan Hound (H/M)	AFGH	1
Boxer* (W/EM)	BOX	8	Beagle or Beagle X (H/SH)	BEAG or BEAG X	4	German Shepherd Dog (P/NW)	GSD	2
Brussels Griffon (To/TS)	BRUS	1	Bearded Collie (P/UR)	BERD	2	Greyhound (H/UR)	GREY	1
Cavalier King Charles Spaniel (To/Sp)	CKCS	18	Bichon Frise (To/Po)	BICH	2	Italian Greyhound (To/UR)	ITGY	1
Chihuahua (To/AT)	CHIH	6	Border Collie (P/UR)	BORD	1	Lurcher or Lurcher X (C/C)	CROS	1
Crossbreed (C/C)	CROS	1	Border Terrier (Te/T)	BORT	4	Miniature Dachshund (H/SH)	DACH	1
Dogue de Bordeaux (W/EM)	DDBX	1	Cockerpoo (C/C)	CROS	2	Weimeraner (G/PS)	WEIM	1
French Bulldog (U/EM)	FBUL	12	Collie or Collie X (P/UR)	COLL or COLL X	1	Whippet* (H/UR)	WHIP or WHIP X	3
King Charles Spaniel (To/Sp)	KCS	2	Crossbreed (C/C)	CROS	4			
Lhasa Apso (U/AsT)	LHSA	1	English Cocker Spaniel (G/Sp)	ECKR	5			
Maltese Terrier (To/Po)	MALT	4	Jack Russell Terrier (Te/T)	JACK or JACK X	4			
Pomeranian (To/SS)	POM	1	Labrador Retriever* (G/R)	LAB	3			
PugDog (To/TS)	PUG	8	Miniature Pinscher (To/P)	MPIN	1			
Shih Tzu (U/AsT)	SHIH	6	Miniature Schnauzer (U/S)	MSNZ	1			
Staffordshire Bull Terrier or X (Te/EM)	STAF or STAF X	5	Parson's Terrier (Te/T)	PARS	1			
Yorkshire Terrier (To/T)	YORK	4	Terrier X (Te/T)	CROS	1			
			West Highland White Terrier (Te/T)	WHWT	4			

Fig.S2. Breed characteristics for MRI scans. (a) MRI scans used in network analysis according to breed (in alphabetical order) with assigned breed abbreviation according to Parker *et al.*,[11] craniofacial category, breed group, and clade. For breed group, C = Crossbreed, G =

Gundog, H = Hound, P = Pastoral, U = Utility, Te = Terrier, To = Toy, W = Working. Yellow = brachycephalic, grey = mesocephalic, green = dolichocephalic. For clade: AT = American Toy, AS = Asian Spitz, AsT = Asian Toy, C = Crossbred, D = Drover, EM = European Mastiff, M = Mediterranean, NW = New World, P = Pinscher, PS = Pointer Setter, Po = Poodle, R = Retriever, SH = Scent Hound, S = Schnauzer, SS = Small Spitz, Sp = Spaniel, T = Terrier, TS = Toy Spitz, UR = UK Rural. 'CROS' is assigned to crossbreed dogs where the parentage was unknown; where information was available for breed contributions, these were specified using the relevant breed abbreviation suffixed with 'X'. Lurcher, Patterdale Terrier, and Cockerpoo are not recognized UK Kennel Club breeds. Nova Scotia DTR = Novia Scotia Duck Tolling Retriever. There were four STAF X scans, three COLL X scans, two JACK X scans, one BEAG X JRT, one WHIP X scan, and one Lurcher X scan. **(b)** MRI scans included in CFR analysis (total 134 scans from 132 dogs). One CROS dog was assigned to the brachycephalic category on the basis of its CFR (0.31). Asterisks refer to breeds for which one representative was scanned twice during the study period.

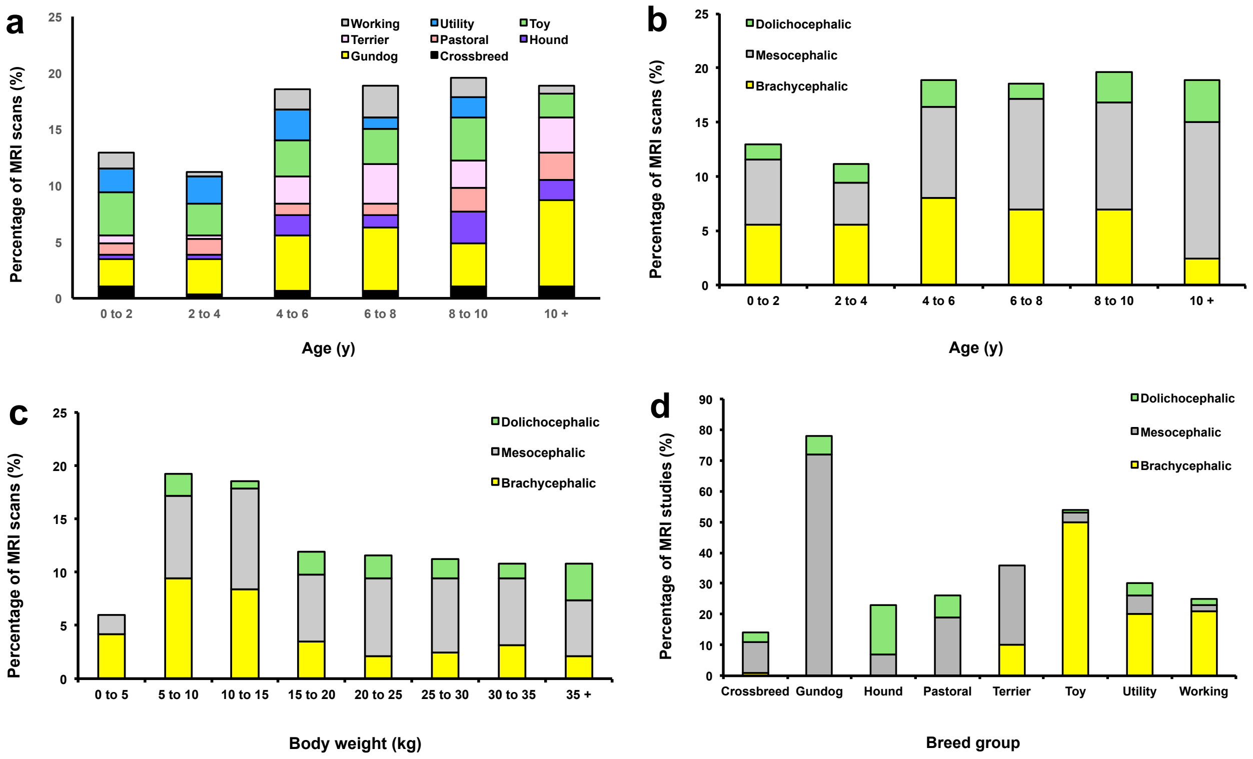

Fig.S3. Age and body weight distribution of dogs in network. Percentage of MRI scans by age according to breed group **(a)**, and craniofacial category **(b)**. **(c)** Percentage of MRI scans by body weight according to craniofacial category. **(d)** Percentage of MRI scans by breed grouping and craniofacial category.

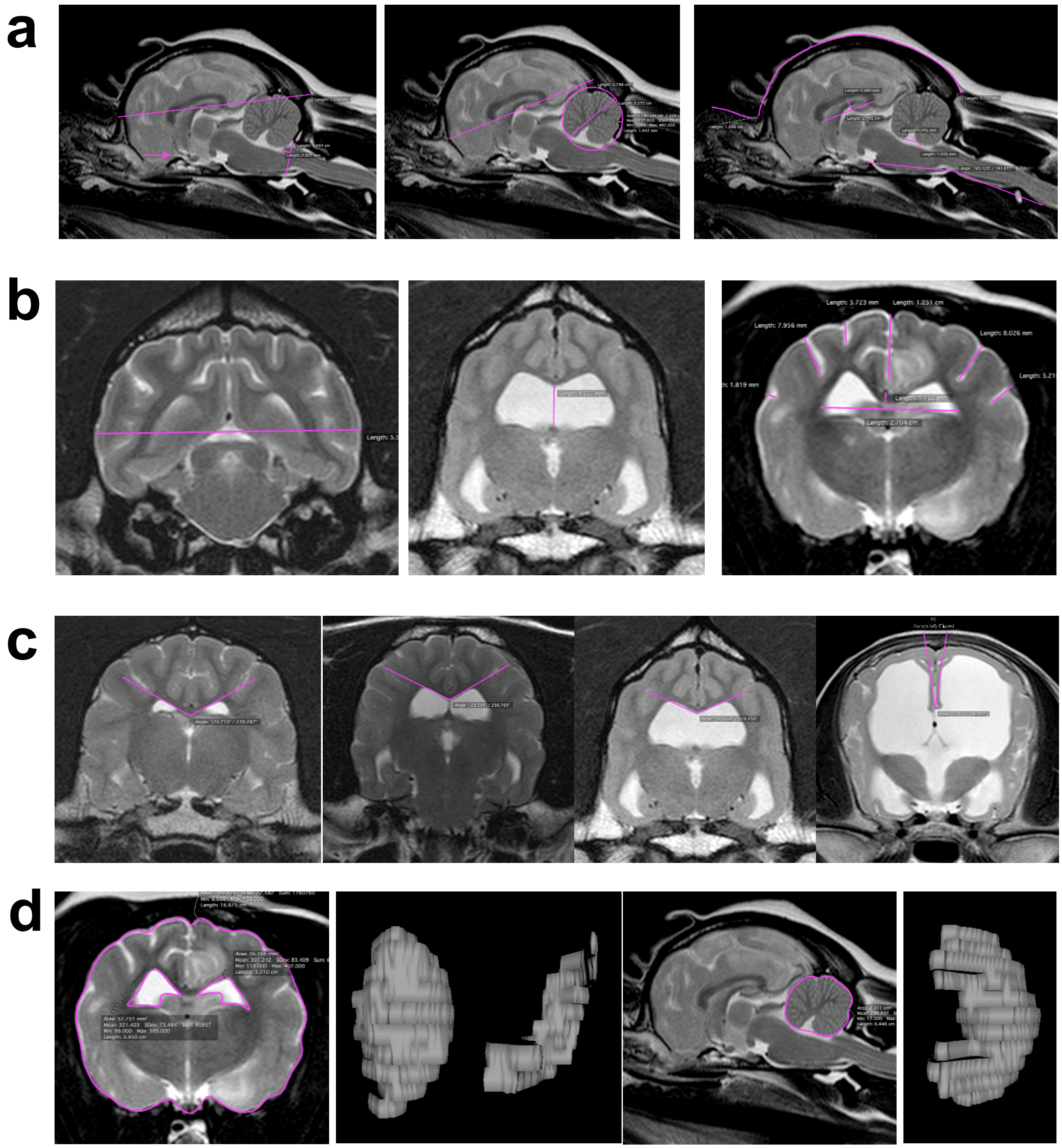

e

Morphometric parameter	Mean % difference between observers	Data input into network
Brain length	0.755	Measured by both observers
Brain width	1.110	Measured by both observers
Cranial length	1.220	Measured by both observers
Total brain volume	2.302	Measured by both observers
Cerebellar volume	32.959	Re-measured by observer B
Ventricle width	16.251	Re-measured by observer B
Interthalamic adhesion height	6.749	Measured by both observers
Callosal height	20.219	Re-measured by observer B
4th ventricle height	27.391	Re-measured by observer B
Width of fastigial recess	27.049	Re-measured by observer B
Ventricular volume (left)	24.139	Re-measured by observer B
Ventricular volume (right)	19.330	Re-measured by observer B
Corpus callosum angle	8.561	Measured by both observers
Corpus callosum thickness	28.045	Re-measured by observer B
Ventricle height	> 10% miscategorized	Re-measured by observer B
Combined sulcus depth	43.748	Re-measured by observer B
Obex position	47.660	Re-measured by observer B
Compression length	24.601	Re-measured by observer B
Cerebellar diameter	5.099	Measured by both observers
Head angle	5.581	Measured by both observers
Cranial length nonlinear	N/A	Measured by observer B
Muzzle length nonlinear	N/A	Measured by observer B

Fig.S4. Canine brain morphometric measurement protocol and reproducibility. (a) Measurements taken on T2-weighted (T2-w) mid-sagittal images included cranial length ('nasion to inion' left), obex position, and height of interthalamic adhesion (left; arrow points to interthalamic adhesion); brain length (cerebrum length), cerebellar diameter (diameter of a circle placed over the widest part of the cerebellum), and cerebellar compression length (distance from the outer limit of the subarachnoid space to the point of greatest neural compression, middle); and craniofacial ratio, callosal height, fourth ventricle height, head angle, and width of fastigial recess (right). Obex position approximated the position of the brainstem by measuring the distance between the caudodorsal-most border of the fourth ventricle and a line drawn parallel to the foramen magnum. Head angle (a quantification of neck flexion) was measured as the angle between a line connecting the dorsum sellae to the caudal basioccipital bone and the floor of the vertebral canal within C2. **(b)** Measurements taken on T2-w transverse magnetic resonance

images included widest width of the brain ('brain width', left); ventricle height (middle; height recorded as zero if septum pellucidum was visually intact); combined sulcus depth, corpus callosum thickness, and widest width of the lateral ventricles or 'ventricle width' (all measured at level of the thickest part of the interthalamic adhesion, right). Corpus callosum thickness was measured at its most ventral midline point. **(c)** The acute corpus callosum angle was also measured on T2-w transverse images at the level of the interthalamic adhesion;[3] the range of ventricular sizes and angulation are highlighted with a dolichocephalic dog (far left), mesocephalic dog (middle left), brachycephalic dog (a Boxer; middle right) and a dog with hydrocephalus (far right). **(d)** Sequential outlines using T2- or T1-w transverse images (for brain and ventricles) and T2-w sagittal images (for cerebellum) were used to compute compartmental volumes. Ventricular asymmetry was derived as the proportion of the left lateral ventricular volume relative to the right. The transverse image (far left) highlights the asymmetry of lateral ventricles in a PugDog, with computed volumes shown for each (left middle and middle images). A 2D representation of the cerebellar volume constructed from sagittal images (right middle) is shown (far right). **(e)** Reproducibility of measurements between observers. Observers were provided only with the unique identifier and date of MRI scan for each dog prior to performing MRI measurements; after data collection was complete, observer B was unblinded to incorporate the morphometric data and linked clinical meta-data into Graphia Professional.

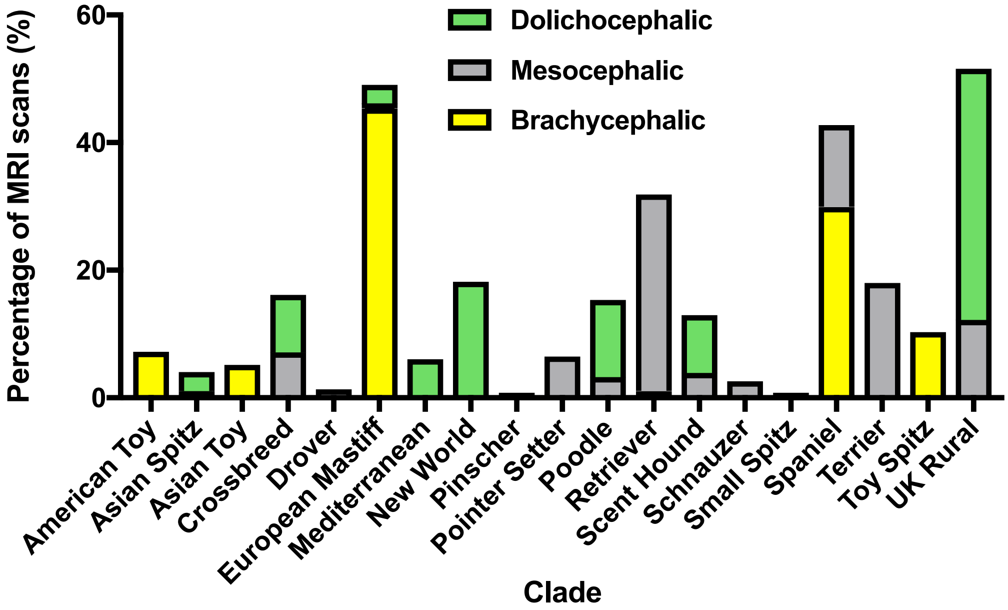

Fig.S5. Percentage of MRI scans by genetic clade. Percentages are coloured according to craniofacial category.

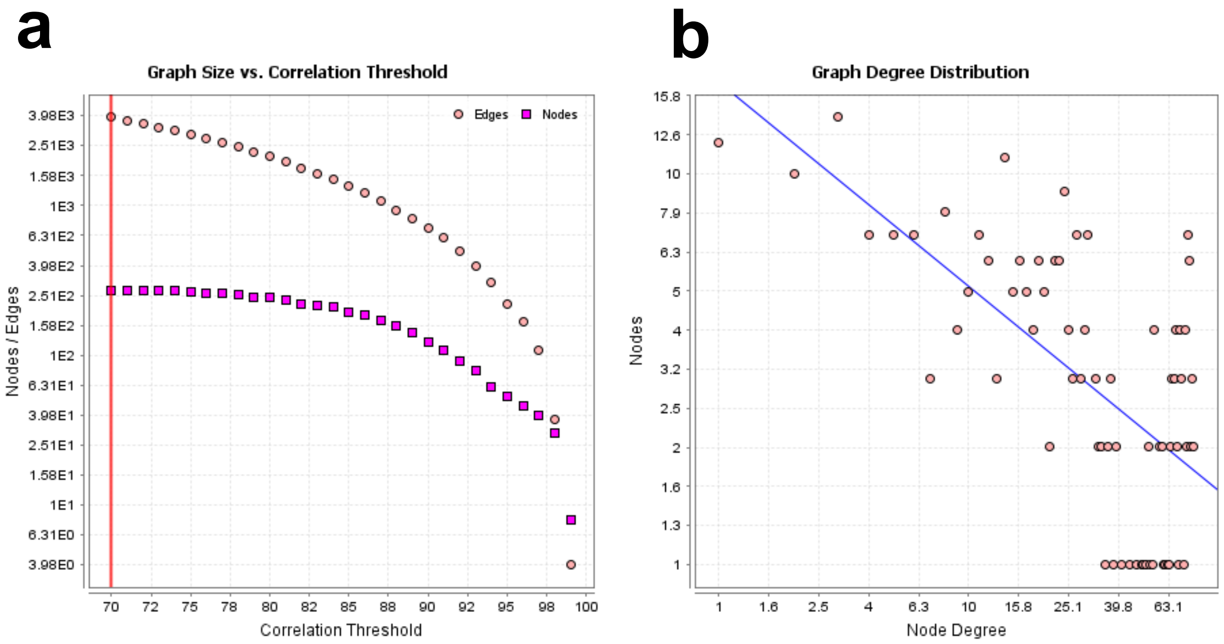

Fig.S6. Correlation graph settings for Graphia Professional data set. (a) Graph size versus correlation threshold, representing the number of nodes and edges incorporated into the network

versus the correlation threshold (set at 0.7). **(b)** Graph degree distribution plotting the number of nodes at each node degree value for the selected threshold.

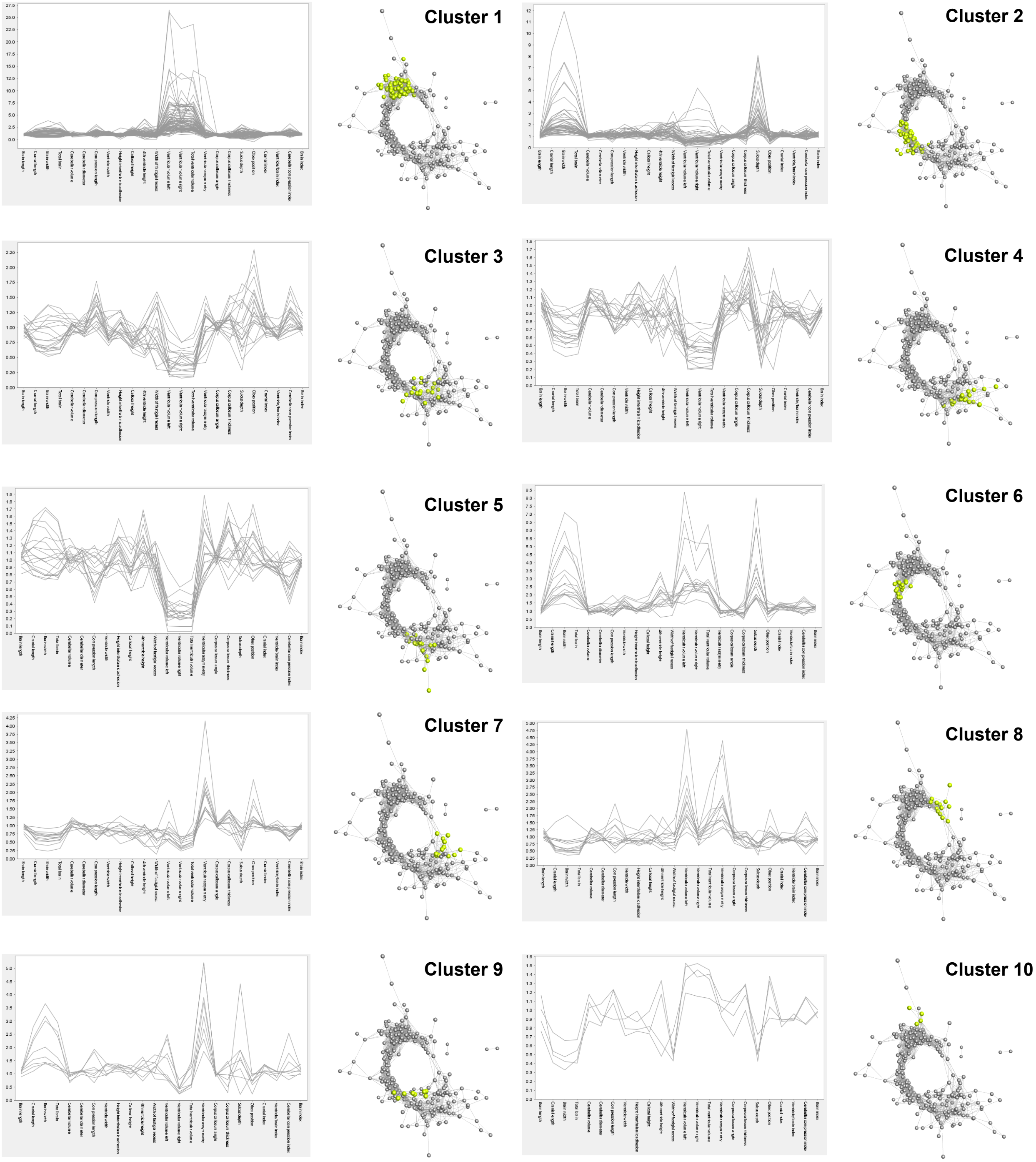

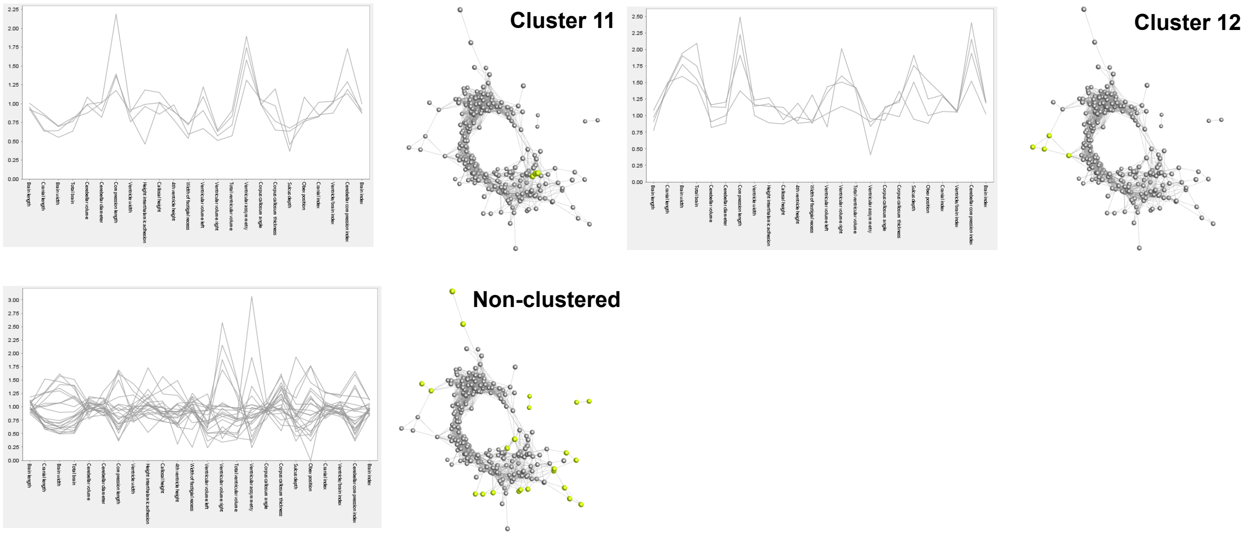

Fig.S7. Cluster characteristics. Clusters one to twelve are highlighted green within each network graph; each MRI scan in each cluster is represented by an individual grey line in the corresponding chart.

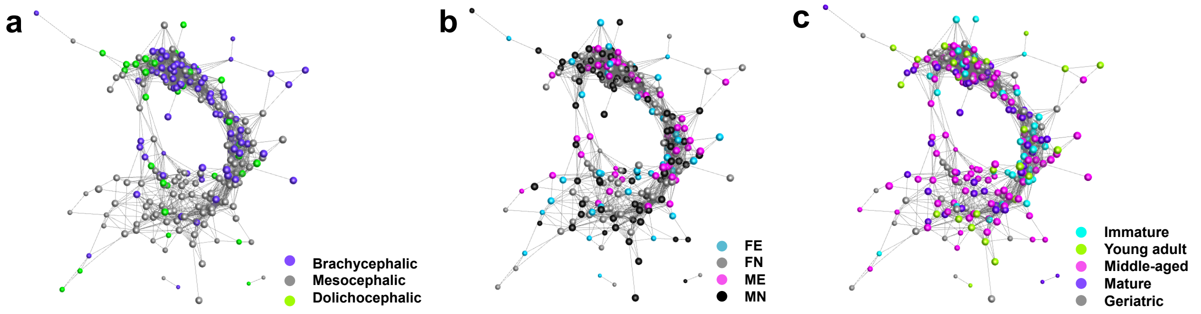

Fig.S8. Networks by signalment. The network is coloured according craniofacial category **(a)**, sex **(b)**, and age category **(c)**.

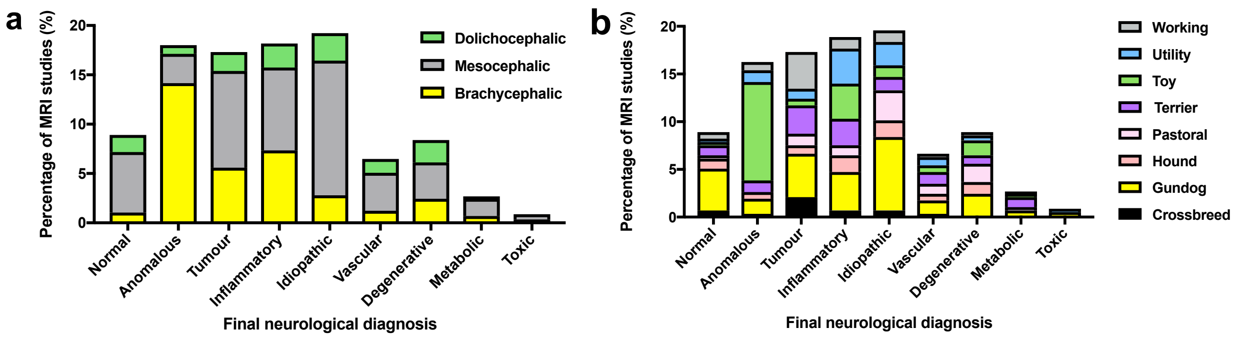

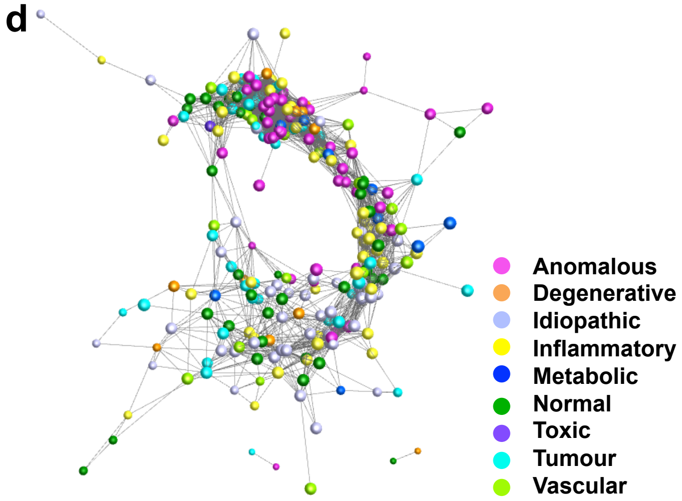

c

Cluster	Descriptor	Observed	Expected	Adj. p-value
1	Tumour	26/86	15.12/86	6.49×10^{-3}
5	Idiopathic	9/18	3.1/18	6.50×10^{-3}
1	Anomalous	25/86	16.07/86	3.39×10^{-2}

Fig.S9. Demographics and enrichment of diagnoses. Percentage of MRI scans by craniofacial category **(a)** and breed group **(b)** according to final diagnosis. **(c)** Enrichment analysis for diagnostic class sets by cluster. Table lists significant enrichments together with expected and observed numbers for each diagnosis in a given cluster, with adjusted *P*-values. For each cluster, diagnoses are listed in order of statistical significance. **(d)** Network coloured according to final neurological diagnosis.

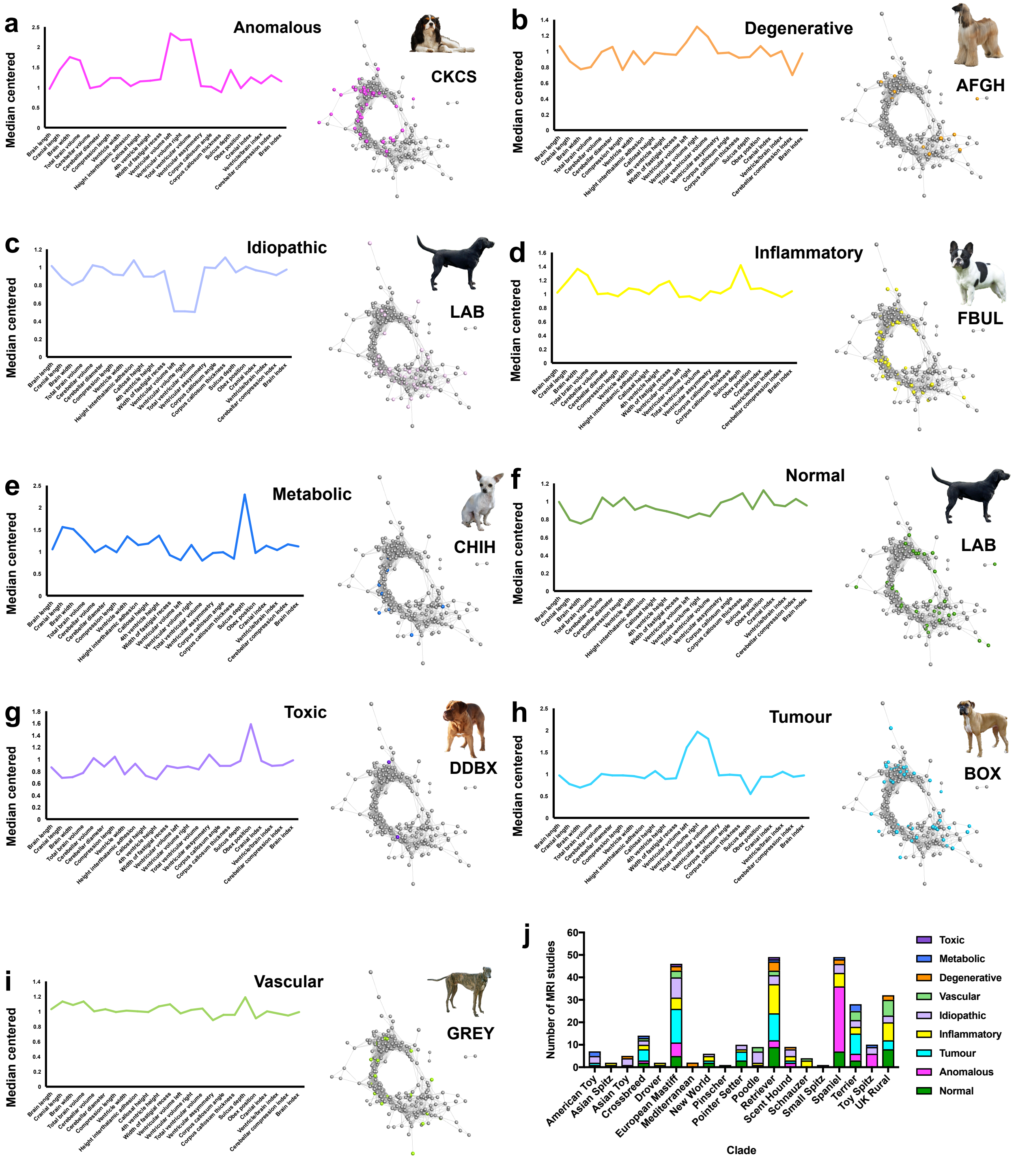

Fig.S10. Diagnostic class morphometrics. Diagnostic classes are coloured as in Figure 3h (**a-i**). In each case, the corresponding 2D chart displays the median MRI scan data. **(j)** Numbers of MRI scans by final neurological diagnosis and clade. Canine breed image attributions: AFGH (By SheltieBoy (Flickr: AKC Helena Fall Dog Show 2011) [CC BY 2.0

(https://creativecommons.org/licenses/by/2.0)]); BOX (By Flickr user boxercab (Flickr here) [CC BY 2.0 (https://creativecommons.org/licenses/by/2.0)]); CKCS (By Mário Simoes (Flickr: Cavalier King Charles Spaniel) [CC BY 2.0 (https://creativecommons.org/licenses/by/2.0)]); CHIH (Photo taken by en:User:Exdumpling in 2004 and uploaded to English Wikipedia as WhiteTanChihuahua.jpg claiming own work with PD-self license); FBUL (By The original uploader was EGILEO at Italian Wikipedia [CC BY 2.0 (https://creativecommons.org/licenses/by/2.0)]; DDBX (By X posid [CC0]); GREY (By FLickr user Scott Feldstein (Flickr here) [CC BY 2.0 (https://creativecommons.org/licenses/by/2.0)]); LAB (By Desaix83, d'après le travail de Chrizwheatley [Public domain]).

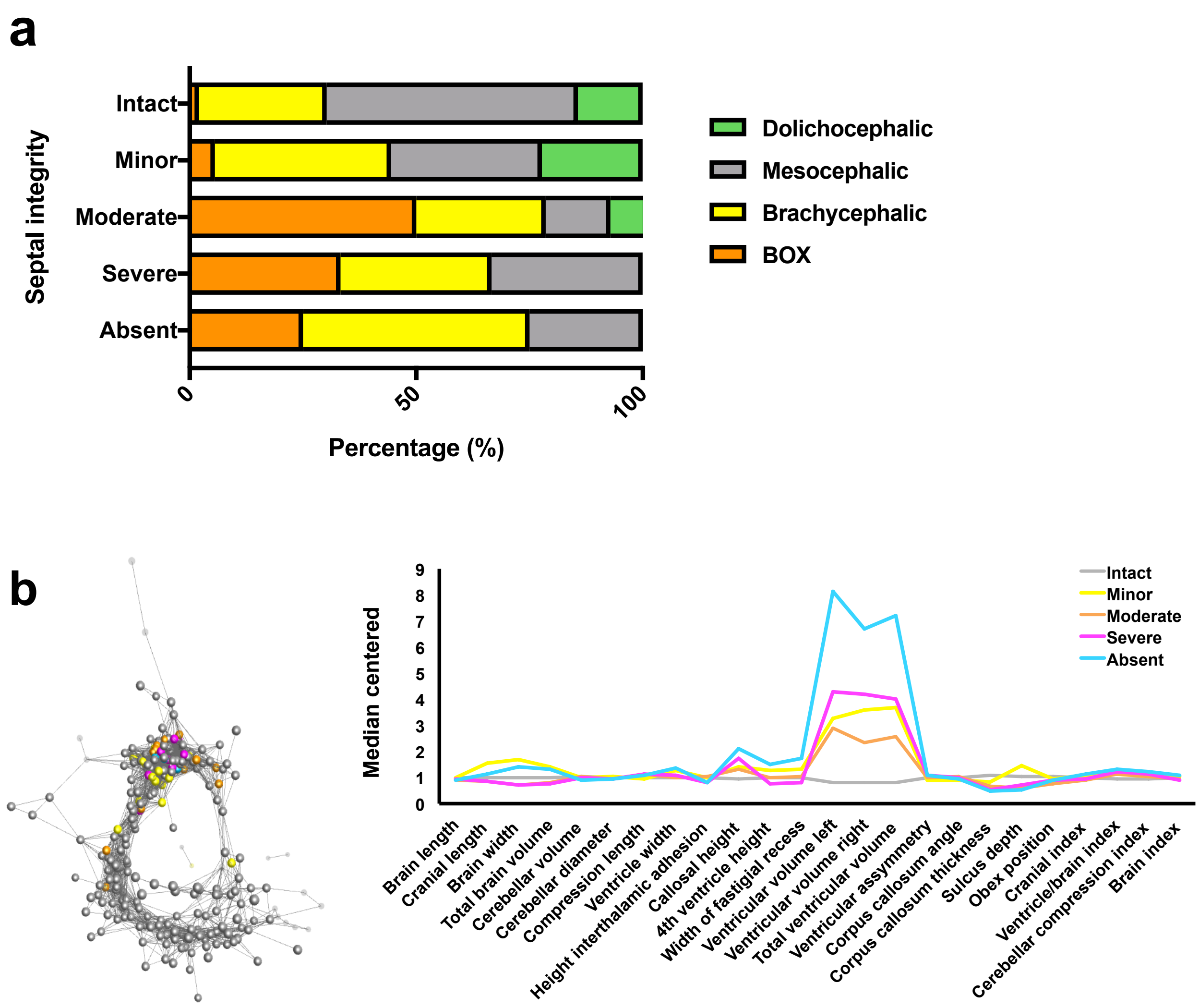

Fig.S11. Septal integrity impacts on brain morphometry. (a) Percentage of MRI scans within each septal integrity class according to craniofacial category. **(b)** Network and chart coloured by septal integrity; MRI scans with compromised septa predominate in cluster one, and ventricular size increases with increasing septal compromise. Blue nodes are buried and include a CKCS, Boxer, PugDog and Crossbreed.

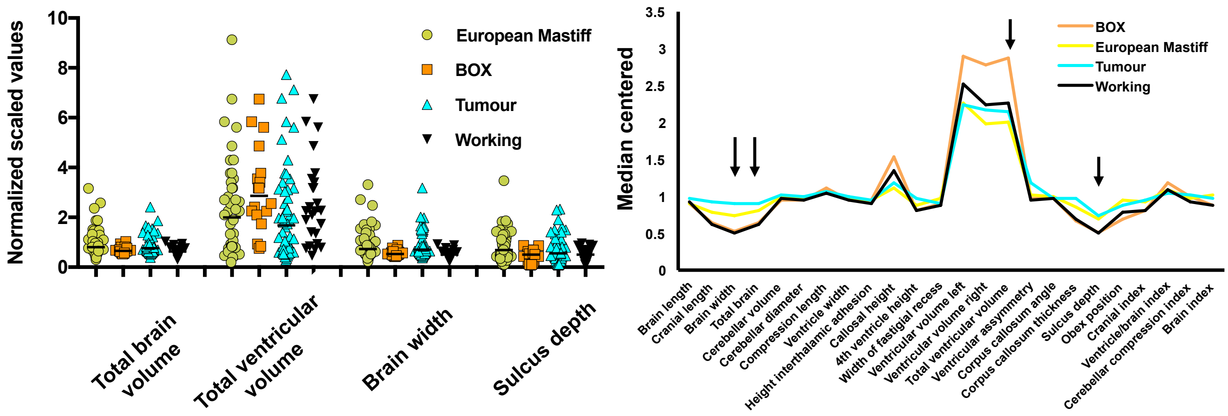

Fig.S12. Shared morphometric features of the Boxer. Key brain morphometric features of the Boxer are shared with other dogs of Working and European Mastiff groups (18/25 and 18/46 were Boxers, respectively), but also with other dogs diagnosed with brain tumours (11/54 were Boxers).

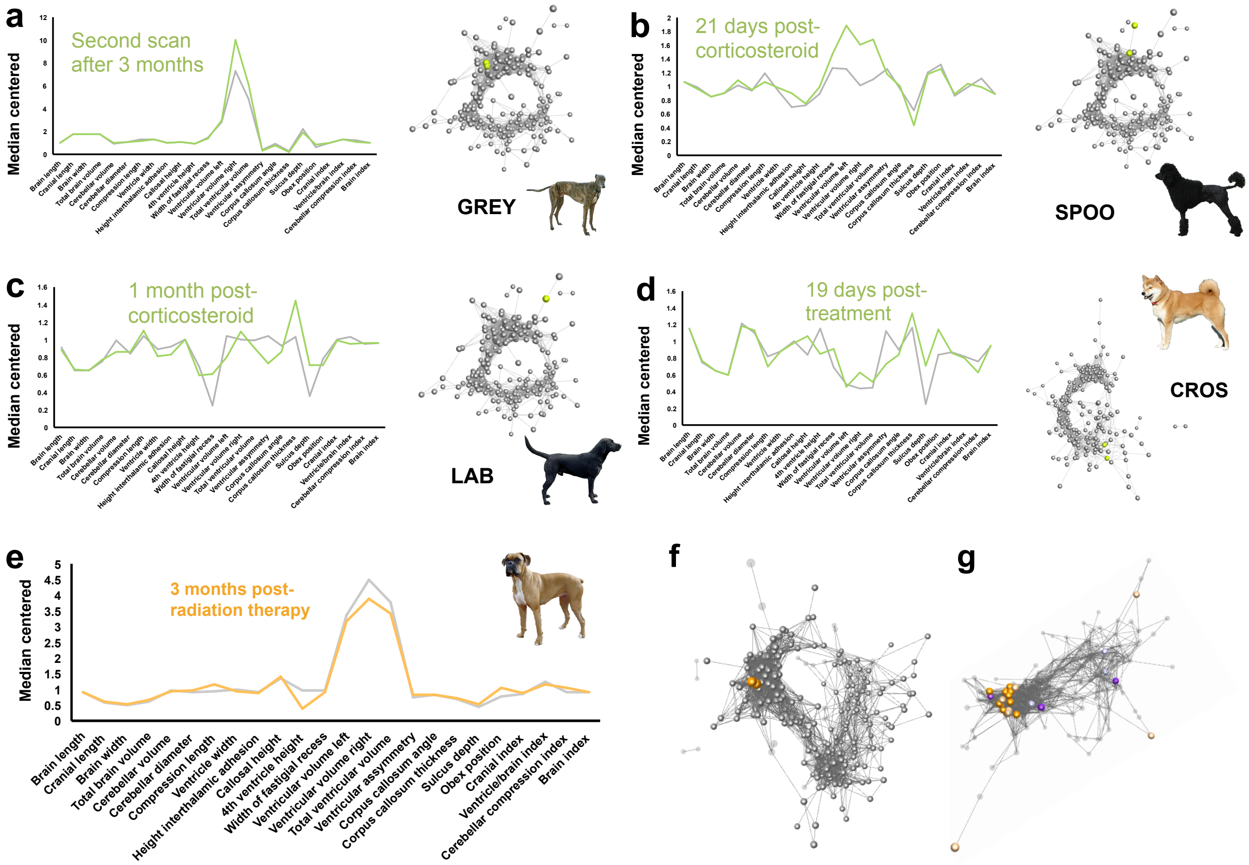

Fig.S13. Patients scanned twice within the study period. (a) A 9y FN Whippet presented with a large cerebrovascular accident (occlusion of right middle cerebral artery). After three months there was persistent vasogenic oedema, secondary cerebral atrophy, and right ventricular distension with a suspected small persistent haemorrhagic component. The morphometric analysis (left) detects the ventricular changes, but the dog remained within cluster one (right). **(b)** A 1y 8m MN Standard Poodle presented with suspected hypophysitis and demonstrated complete macroscopic resolution in response to three weeks of corticosteroid treatment.[10] Note predominant shift in ventricular parameters and interthalamic adhesion height (left) but retention of dog within cluster one (right). **(c)** A 10y FE Labrador Retriever presented with an intra-axial inflammatory mass lesion that resolved macroscopically after one month of corticosteroid treatment. Note marked shifts in morphometry of several fluid-filled spaces (left), sufficient to displace the dog from the non-clustered grouping out of the network entirely (only one node highlighted on right). **(d)** A 7y 9m MN Akita X Boxer dog presented with a suspected glioma between the right parietal lobe and cingulate gyrus. There was marked but incomplete response

of this lesion to corticosteroid treatment; after 19 days sulcus depth had increased, and ventricular parameters and obex position had shifted (left), although the dog remained in cluster four (right). In each case the network has been rotated to best visualize the scans of interest. **(e-f)** A 5y 4m FN Boxer with a suspected oligodendroglioma that demonstrated macroscopic resolution in response to radiation therapy. Morphometric analysis showed a small reduction in fourth ventricle height, and shift in obex position and ventricular volume, but retention of dog within cluster 1 (orange nodes in **(g)**). **(h)** highlights the patients compared in Figure 4e; orange nodes = Boxers with tumours, pale orange nodes = Boxers without tumours, purple nodes = Weimeraners with tumours, lilac nodes = Weimeraners without tumours. Canine breed image attributions: BOX (By Flickr user boxercab (Flickr here) [CC BY 2.0 (https://creativecommons.org/licenses/by/2.0)]); GREY (By FLickr user Scott Feldstein (Flickr here) [CC BY 2.0 (https://creativecommons.org/licenses/by/2.0)]); LAB (By Desaix83, d'après le travail de Chrizwheatley [Public domain]); Shiba Inu (By Takashiba at English Wikipedia [Public domain]); SPOO (By Inbalsigal [CC BY 3.0 (https://creativecommons.org/licenses/by/3.0)]).

Class	Descriptor	Observed	Expected	Adj. p-value
FE	Immature	14/41	5.56/41	8.97×10^{-4}
	Intact	40/41	33.79/41	6.50×10^{-3}
	American Toy	5/41	1.05/41	1.22×10^{-2}
	CHIH	5/41	1.05/41	2.34×10^{-2}
ME	FBUL	7/52	1.71/52	4.03×10^{-3}
	Utility	13/52	5.52/52	5.28×10^{-3}
	Brachycephalic	27/52	18.86/52	2.12×10^{-2}
	European Mastiff	16/52	8.19/52	2.63×10^{-2}

Fig.S14. Enrichments by sex. All statistically significant enrichments for sex classes are shown. Note the strong enrichment of immature morphometry profiles within the un-neutered female class.

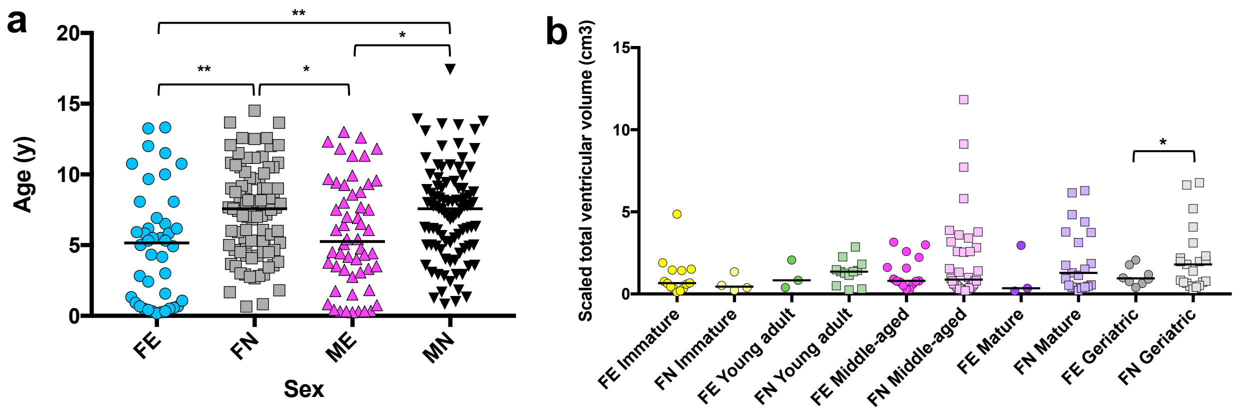

Fig.S15. Ventricular enlargement in neutered female dogs (a) Neutered animals were significantly older within the cohort ($N = 286$, $P = 0.0002$; one-way ANOVA); asterisks refer to multiplicity adjusted P-values by Tukey's method (**$P < 0.01$, *$P < 0.05$). **(b)** Controlling for age, the trend is for enhanced ventricular enlargement with increasing age in neutered versus un-neutered females, reaching significance in the geriatric group ($N = 29$, *$P = 0.0365$; two-tailed unpaired t-test with Welch's correction).

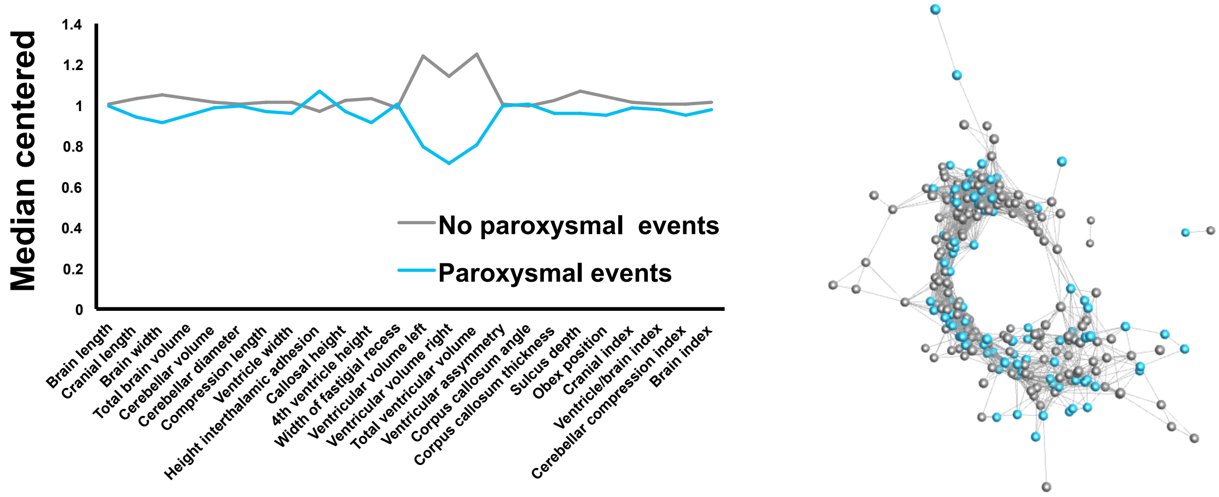

Fig.S16. Dichotomous brain morphometry in dogs with and without paroxysmal events. Morphometric differences are revealed in the chart (left) for dogs with and without a history of paroxysmal events (generalized seizures, partial seizures, general and partial seizures, paroxysmal dyskinesia, and 'drop attack'-like paroxysms). Note that both groups are spread evenly across the network (right), but their brain morphometries diverge with respect to interthalamic adhesion height, whole brain parameters, and ventricular size.

Cluster	Breed	Breed type	Breed function	Clade	Final neurological diagnosis	Septal integrity	Sex	Age	Paroxysmal events
**1 (86/83)**	BOX 15/14<br>CKCS 13<br>LAB 7<br>PUG 5<br>OTHER 46/44	Brachycephalic 44/43<br>Mesocephalic 31<br>Dolichocephalic 11/9	Toy 21<br>Working 17<br>Gundog 16<br>Terrier 10<br>Hound 9/8<br>Utility 7/6<br>Pastoral 4<br>Crossbreed 2	European Mastiff 24/23<br>Spaniel 17<br>Retriever 11<br>Terrier 7<br>Toy Spitz 6<br>Scent Hound 5<br>UK Rural 5/4<br>Poodle 3/2<br>Pointer Setter 2<br>Crossbreed 2<br>Asian Spitz 1<br>Drover 1<br>New World 1<br>Mediterranean 1	Tumour 26/25<br>Anomalous 25<br>Inflammatory 13/12<br>Degenerative 6<br>Normal 5<br>Vascular 5/4<br>Idiopathic 4<br>Metabolic 2	Intact 50/49<br>Minor 13/12<br>Severe 11<br>Moderate 8/7<br>Absent 4	FN 32/30<br>MN 30/29<br>FE 5<br>ME 19	Middle-aged 30/29<br>Mature 22/21<br>Geriatric 19<br>Young adult 8<br>Immature 7/6	N 64/63<br>G 18/17<br>DA 2<br>P 2/1
**2 (46/46)**	CHIH 5<br>FBUL 5<br>ECKR 4<br>SHIH 3<br>JACK 3<br>BORD 3<br>OTHER 23	Mesocephalic 23<br>Brachycephalic 18<br>Dolichocephalic 5	Utility 12<br>Toy 12<br>Terrier 9<br>Crossbreed 4<br>Gundog 4<br>Pastoral 3<br>Hound 2	Terrier 9<br>European Mastiff 6<br>UK Rural 6<br>American Toy 5<br>Crossbreed 4<br>Poodle 4<br>Spaniel 4<br>Asian Toy 3<br>Schnauzer 2<br>Pinscher 1<br>Small Spitz 1<br>Toy Spitz 1	Inflammatory 13<br>Normal 8<br>Idiopathic 8<br>Anomalous 5<br>Vascular 5<br>Metabolic 4<br>Tumour 3	Intact 43<br>Moderate 2<br>Minor 1	MN 15<br>ME 13<br>FE 9<br>FN 9	Immature 14<br>Middle-aged 14<br>Mature 9<br>Geriatric 5<br>Young adult 4	N 31<br>G 9<br>P 3<br>PD 3
**3 (20/20)**	LAB 8<br>ESSP 4<br>CKCS 2<br>OTHER 6	Mesocephalic 17<br>Brachycephalic 3	Gundog 14<br>Toy 2<br>Utility 2<br>Hound 1<br>Pastoral 1	Retriever 10<br>Spaniel 7<br>European Mastiff 1<br>Scent Hound 1<br>UK Rural 1	Idiopathic 7<br>Normal 5<br>Inflammatory 3<br>Anomalous 2<br>Degenerative 1<br>Toxic 1<br>Tumour 1	Intact 20	FN 8<br>ME 5<br>FE 4<br>MN 3	Middle-aged 9<br>Mature 5<br>Immature 3<br>Young adult 2<br>Geriatric 1	N 12<br>G 6<br>DA 1<br>PD 1
**4 (19/18)**	LAB 4<br>ESSP 3<br>CROS 2<br>OTHER 11/10	Mesocephalic 16/15<br>Brachycephalic 2<br>Dolichocephalic 1	Gundogs 9<br>Crossbreed 4/3<br>Pastoral 3<br>Terrier 1<br>Utility 1<br>Working 1	Retriever 5<br>Crossbreed 4/3<br>Pointer Setter 3<br>Spaniel 3<br>UK Rural 3<br>European Mastiff 1	Idiopathic 6<br>Normal 4<br>Tumour 4/3<br>Inflammatory 3<br>Vascular 2	Intact 19/18	MN 8/7<br>FN 7<br>FE 3<br>ME 1	Middle-aged 8/7<br>Geriatric 4<br>Mature 3<br>Young adult 3<br>Immature 1	N 13/12<br>G 5<br>PD 1
**5 (18/18)**	ECKR 4<br>BORD 3<br>OTHER 11	Mesocephalic 16<br>Brachycephalic 2	Gundog 7<br>Pastoral 5<br>Utility 3<br>Terrier 2<br>Crossbreed 1	Spaniel 5<br>UK Rural 5<br>Retriever 2<br>Schnauzer 2<br>Asian Spitz 1<br>Crossbreed 1<br>European Mastiff 1<br>Terrier 1	Idiopathic 9<br>Inflammatory 3<br>Anomalous 2<br>Degenerative 1<br>Normal 1<br>Tumour 1<br>Vascular 1	Intact 18	MN 9<br>FN 8<br>ME 1	Middle-aged 7<br>Young adult 4<br>Mature 3<br>Geriatric 2<br>Immature 2	N 9<br>G 7<br>P 1<br>PD 1
**6 (15/15)**	CKCS 3<br>JACK 2<br>MALT 2<br>PUG 2	Brachycephalic 11<br>Mesocephalic 3<br>Dolichocephalic 1	Toy 9<br>Terrier 3<br>Utility 2<br>Pastoral 1	Terrier 4<br>Spaniel 3<br>Poodle 2<br>Toy Spitz 2<br>American Toy 1<br>Asian Toy 1<br>European Mastiff 1<br>UK Rural 1	Anomalous 6<br>Inflammatory 4<br>Vascular 2<br>Degenerative 1<br>Idiopathic 1<br>Metabolic 1	Intact 11<br>Minor 2<br>Moderate 1<br>Severe 1	FN 6<br>FE 4<br>ME 4<br>MN 1	Middle-aged 7<br>Geriatric 2<br>Immature 2<br>Mature 2<br>Young adult 1	N 8<br>G 6<br>P 1
**7 (13/13)**	LAB 4<br>WEIM 2<br>OTHER 7	Mesocephalic 7<br>Brachycephalic 4<br>Dolichocephalic 2	Gundog 8<br>Terrier 2<br>Hound 1<br>Utility 1<br>Working 1	Retriever 5<br>European Mastiff 4<br>Pointer Setter 3<br>Scent Hound 1	Tumour 4<br>Idiopathic 2<br>Normal 2<br>Inflammatory 2<br>Degenerative 1<br>Metabolic 1<br>Vascular 1	Intact 12<br>Minor 1	ME 4<br>FE 3<br>FN 3<br>MN 3	Middle-aged 7<br>Geriatric 3<br>Mature 3	N 9<br>G 4
**8 (13/13)**	LAB 4<br>GSD 2<br>OTHER 7	Dolichocephalic 6<br>Mesocephalic 6<br>Brachycephalic 1	Gundog 6<br>Pastoral 3<br>Hound 1<br>Utility 1<br>Working 1<br>Crossbreed 1	Retriever 5<br>New World 2<br>UK Rural 2<br>Asian Toy 1<br>Crossbreed 1<br>European Mastiff 1<br>Pointer Setter 1	Normal 4<br>Inflammatory 3<br>Idiopathic 2<br>Tumour 2<br>Anomalous 1<br>Vascular 1	Intact 10<br>Moderate 3	MN 8<br>FE 3<br>FN 2	Middle-aged 5<br>Geriatric 2<br>Immature 2<br>Mature 2<br>Young adult 2	N 11<br>G 2
**9 (8/8)**	JACK 2<br>OTHER 6	Mesocephalic 4<br>Brachycephalic 3<br>Dolichocephalic 1	Terrier 3<br>Toy 3<br>Gundog 1<br>Pastoral 1	Terrier 3<br>Spaniel 2<br>American Toy 1<br>New World 1<br>Toy Spitz 1	Anomalous 3<br>Idiopathic 2<br>Tumour 1<br>Inflammatory 1<br>Vascular 1	Intact 8	FE 3<br>MN 3<br>FN 1<br>ME 1	Middle-aged 4<br>Immature 3<br>Geriatric 1	N 5<br>P 2<br>G 1
**10 (4/4)**	OTHER 4	Mesocephalic 2<br>Brachycephalic 1<br>Dolichocephalic 1	Gundog 2<br>Pastoral 1<br>Working 1	Retriever 2<br>European Mastiff 1<br>New World 1	Anomalous 1<br>Inflammatory 1<br>Normal 1<br>Toxic 1	Intact 4	FN 2<br>ME 1<br>MN 1	Young adult 2<br>Immature 1<br>Mature 1	N 3<br>G 1
**11 (4/4)**	OTHER 4	Dolichocephalic 2<br>Brachycephalic 1<br>Mesocephalic 1	Hound 2<br>Gundog 1<br>Working 1	European Mastiff 1<br>Retriever 1<br>Scent Hound 1<br>UK Rural 1	Tumour 2<br>Degenerative 1<br>Idiopathic 1	Intact 4	FN 2<br>MN 2	Geriatric 3<br>Young adult 1	G 2<br>N 1<br>P 1
**12 (4/4)**	CKCS 2<br>KCS 1<br>CROS 1	Brachycephalic 3<br>Mesocephalic 1	Toy 3<br>Terrier 1	Spaniel 3<br>Terrier 1	Anomalous 2<br>Normal 1<br>Tumour 1	Intact 4	FN 2<br>ME 1<br>MN 1	Young adult 2<br>Geriatric 1<br>Middle-aged 1	N 4
**No class (23/23)**	LAB 5<br>CKCS 4<br>GREY 3<br>CROS 3<br>OTHER 8	Mesocephalic 12<br>Brachycephalic 6<br>Dolichocephalic 5	Gundog 7<br>Hound 4<br>Toy 4<br>Terrier 3<br>Working 3<br>Pastoral 1<br>Crossbreed 1	Retriever 6<br>Spaniel 5<br>UK Rural 5<br>Terrier 3<br>European Mastiff 2<br>Drover 1<br>Crossbreed 1	Idiopathic 5<br>Tumour 3<br>Anomalous 4<br>Normal 4<br>Degenerative 2<br>Inflammatory 3<br>Metabolic 1<br>Vascular 1	Intact 22<br>Minor 1	FN 8<br>FE 7<br>MN 6<br>ME 2	Middle-aged 9<br>Geriatric 7<br>Mature 3<br>Immature 2<br>Young adult 2	N 11<br>G 9<br>PD 2<br>GP 1

Table S1. Clusters generated for network. ME = entire male, MN = neutered male, FE = entire female, FN = neutered female. N = no paroxysmal events, G = generalized seizures, P = partial seizures, GP = generalized and partial seizures, PD = paroxysmal dyskinesia, DA 'drop attack'-like paroxysms. 'Other' refers to a combination of breeds where there were fewer representatives within the cluster.

Supplementary References

1. Parker, H.G. et al. Genomic Analyses Reveal the Influence of Geographic Origin, Migration, and Hybridization on Modern Dog Breed Development. *Cell Rep*. **19,** 697708 (2017).
2. Schmidt, M.J., Amort, K.H., Failing, K., Klingler, M., Kramer, M. & Ondreka, N. Comparison of the endocranial- and brain volumes in brachycephalic dogs, mesaticephalic dogs and Cavalier King Charles Spaniels in relation to their body weight. *Acta Vet. Scand*. **56,** 30 (2014).
3. Harcourt-Brown, T., Campbell, J., Warren-Smith, C., Jeffery, N.D. & Granger, N.P. Prevalence of Chiari-like malformations in clinically unaffected dogs. *J. Vet. Intern. Med*. **29,** 231–237 (2015).
4. Driver, C.J., Volk, H.A., Rusbridge, C. & Van Ham, L.M. An update on the pathogenesis of syringomyelia secondary to Chiari-like malformations in dogs. *Vet. J*. **198,** 551–559 (2013).
5. MacKillop, E. Magnetic resonance imaging of intracranial malformations in dogs and cats. *Vet. Radiol. Ultrasound* **52,** S42–S51 (2011).
6. Laubner, S., Ondreka, N., Failing, K., Kramer, M. & Schmidt, M.J. Magnetic resonance imaging signs of high intraventricular pressure--comparison of findings in dogs with clinically relevant internal hydrocephalus and asymptomatic dogs with ventriculomegaly. *BMC Vet. Res*. **11,** 181 (2015).
7. Ryan, C.T., Glass, E.N., Seiler, G., Zwingenberger, A.L. & Mai, W. Magnetic resonance imaging findings associated with lateral cerebral ventriculomegaly in English bulldogs. *Vet. Radiol. Ultrasound* **55,** 292–299 (2014).

8. Cerda-Gonzalez, S., Olby, N.J. & Griffith, E.H. Dorsal compressive atlantoaxial bands and the craniocervical junction syndrome: association with clinical signs and syringomyelia in mature Cavalier King Charles Spaniels. *J. Vet. Intern. Med.* **29,** 887–892 (2015).
9. Marino, D.J. et al. Morphometric features of the craniocervical junction region in dogs with suspected Chiari-like malformation determined by combined use of magnetic resonance imaging and computed tomography. *Am. J. Vet. Res.* 2012; **73:** 105–111.
10. Rzechorzek, N.M., Liuti, T., Stalin, C. & Marioni-Henry, K. Restored vision in a young dog following corticosteroid treatment of presumptive hypophysitis. *BMC Vet. Res.* **13,** 63 (2017).
11. Driver, C.J., Chandler, K., Walmsley, G., Shihab, N. & Volk, H.A. The association between Chiari-like malformation, ventriculomegaly and seizures in Cavalier King Charles Spaniels. *Vet. J.* **195,** 235–7 (2013).
